# Early diagnostic and prognostic biomarkers for gastric cancer: systems-level molecular basis of subsequent alterations in gastric mucosa from chronic atrophic gastritis to gastric cancer

**DOI:** 10.1186/s43141-023-00539-0

**Published:** 2023-08-18

**Authors:** Tamizh G. Selvan, Pavan Gollapalli, Santosh H. S. Kumar, Sudeep D. Ghate

**Affiliations:** 1 Central Research Laboratory, K S Hegde Medical Academy, Nitte (Deemed to Be University), Deralakatte, Mangalore, 575018 Karnataka India; 2 Center for Bioinformatics, University Annexe, Nitte (Deemed to be University), Deralakatte, Mangalore, 575018 Karnataka India; 3 Department of Biotechnology, Jnana Sahyadri Campus, Kuvempu University, Shankaraghatta, 577451 Karnataka India

**Keywords:** Chronic atrophic gastritis, Gastric cancer, Systems biology, MCM7, Functional enrichment

## Abstract

**Purpose:**

It is important to comprehend how the molecular mechanisms shift when gastric cancer in its early stages (GC). We employed integrative bioinformatics approaches to locate various biological signalling pathways and molecular fingerprints to comprehend the pathophysiology of the GC. To facilitate the discovery of their possible biomarkers, a rapid diagnostic may be made, which leads to an improved diagnosis and improves the patient’s prognosis.

**Methods:**

Through protein–protein interaction networks, functional differentially expressed genes (DEGs), and pathway enrichment studies, we examined the gene expression profiles of individuals with chronic atrophic gastritis and GC.

**Results:**

A total of 17 DEGs comprising 8 upregulated and 9 down-regulated genes were identified from the microarray dataset from biopsies with chronic atrophic gastritis and GC. These DEGs were primarily enriched for CDK regulation of DNA replication and mitotic M-M/G1 phase pathways, according to KEGG analysis (*p* > 0.05). We discovered two hub genes, *MCM7* and *CDC6*, in the protein–protein interaction network we obtained for the 17 DEGs (expanded with increased maximum interaction with 110 nodes and 2103 edges). *MCM7* was discovered to be up-regulated in GC tissues following confirmation using the GEPIA and Human Protein Atlas databases.

**Conclusion:**

The elevated expression of *MCM7* in both chronic atrophic gastritis and GC, as shown by our comprehensive investigation, suggests that this protein may serve as a promising biomarker for the early detection of GC.

**Supplementary Information:**

The online version contains supplementary material available at 10.1186/s43141-023-00539-0.

## Background

One of the most frequent and dangerous malignancies in the world, particularly among elderly men, is gastric cancer (GC). Based on WHO data, GC is the 6th most common neoplasm (1.09 million cases) and the 4th most lethal cancer [1]. The mucous membrane lining the stomach comprises columnar epithelial cells and glands. These cells are prone to gastritis, an inflammation that can develop into peptic ulcers and, eventually, stomach cancer. GC is thought to be preceded by chronic atrophic gastritis (CAG). Although the actual aetiology of atrophic gastritis is uncertain, *Helicobacter pylori (H. pylori*) bacteria are recognised to be the most common cause [2].

Normal stomach epithelium is the first step in the Correa cascade of gastric carcinogenesis, which progresses through chronic non-atrophic gastritis, CAG, intestinal metaplasia (IM), and dysplasia [3]. CAG is frequently brought on by anomalies in a number of signalling pathways, including those that regulate apoptosis, the immune system, and inflammation. The signal will transmit extracellular information into the cell as it is exposed to external stimuli, causing the transcription of the right target genes and regulating cell activity [4]. It is necessary to modify signal transduction pathways to prevent GC or reverse CAG. Premalignant gastric lesions (CAG, IM, or dysplasia) enhance the likelihood of developing GC in a person. Early diagnosis of these conditions is essential for successful treatment and GC screening [5]. The clinical diagnosis of GC has developed in recent years, with promising biomarkers such as E-cadherin, p27, HER2, cyclin E, c-myc, and p53 [6]. The diagnosis of GC is made using invasive methods, such as endoscopic ultrasound screening, computed tomography, magnetic resonance imaging, and gastroscopy with biopsy and histological analysis [7]. Additionally, the concentrations of biochemical tumour markers including the carcinoembryonic antigen (CEA), carbohydrate antigen (CA19-9), and cancer antigen 72–4 (CA72-4) are crucial in the diagnosis of patients with this malignancy, but they cannot be utilised to identify GC early [8].

In recent years, biomarkers linked with tumour development, diagnosis, and prognosis have been discovered using several bioinformatics methodologies [9–12]. However, review of accessible literature indicated that no biomarkers have been identified that can be used to predict the progression of CAG to GC. Novel blood biomarkers are therefore required to enhance the diagnostic process, particularly the early detection of this disease, increase the likelihood of successful therapy, and increase the number of cancer survivors. Understanding the changes in molecular pathways that occur during the early stages of GC development and identifying relevant biomarkers can lead to a faster diagnosis and a better prognosis for patients [13]. The molecular mechanism that leads to the progression of CAG to GC is unknown. Identifying genes linked to GC development and prognosis, as well as elucidating the underlying molecular pathways, is critical. Through bioinformatic analysis of Gene Expression Omnibus (GEO) datasets, we aimed to identify putative pathogenic and prognostic differentially expressed genes (DEGs) in CAG that resulted in GC. A pipeline starting with an analysis of DEG from GEP dataset followed by functional enrichment and subsequent cross verification by multiple dataset analysis has helped us develop potentially unique and specific diagnostic biomarkers.

## Methods

### Microarray data collection, pre-processing, and differentially expressed gene extraction

The Gene Expression Omnibus (http://www.ncbi.nlm.nih.gov/geo/) database was used to retrieve the microarray data (accession number: GSE116312) based on the platform of [HuGene-1 0-st] Affymetrix Human Gene 1.0 ST Array [transcript (gene) version]. RNA from biopsies of patients (*n* = 13) with CAG, follicular gastritis (FG), and GC was examined using microarrays. The data set comprised seven FG biopsy samples, three CAG biopsy samples, and three GC biopsy samples.

The data was presented using a gene expression matrix. To transfer the probe data to a gene annotation file, the gene mean value in various samples has to be distributed uniformly across all samples. If multiple probes were matched for a gene, the average of all probe results would represent the gene’s expression. The missing value was located using the k-Nearest Neighbour function of the R impute package (https://bioconductor.org/biocLite.R). The limma package in Bioconductor R (https://bioconductor.org/biocLite.R) was used to identify genes that were differentially expressed between CAG-GC, CAG-FG, and FG-GC. The log2fold change was estimated (log2FC). The cut-off values for the DEGs screening were |log2FC|> 2 and a false discovery rate (FDR) of 0.05. In order to analyse biological pathways, interaction network enrichment analysis, and gene functional annotation, these DEGs will be used.

### DEGs intersection and common DEGs finding

The data mining technique is employed to locate eligible data and common genes among CAG, FG, and GC. The couplings between CAG-FG, FG-GC, and CAG-GC are studied to determine the intersection of genes between/among these three disorders. The intersection result can be used to guide future study and identify shared genes. The final shared gene between CAG-FG-GC facilitates effective biomarker discovery and/or drug design.

### Construction of protein–protein interaction (PPI) network

The STRING (Search Tool for the Retrieval of Interacting Genes) database is a pre-computed global resource for assessing PPI data [14]. The PPIs comprise a vast and complex regulatory network that has been linked to numerous physiological and pathological processes [15]. The edges of the PPI network show interactions between nodes, and each node in the network represents a gene. High-degree nodes are categorised as hub genes with significant biological functions since they have a large number of edges connecting them to other nodes. In this study, the PPI network of common DEGs was analysed using the STRING online tool. Using cystoscope 3.9.1 [16], interactions of common DEGs with a confidence score of > 0.4 and a maximum number of interactions in the first shell as 100 were chosen for research. The PPI network’s genes were further examined in terms of degree centrality, betweenness centrality (BC), and subgraph centrality using the Network Analyzer [17].

### Identification of key genes by centralities based topological analysis of the protein interaction network (PIN)

A network of nodes with varying degrees of connectivity can be used to illustrate the molecular organisation. A protein is represented by each node, and the edges denote dynamic interactions. As a result, nodes get input and output values from mathematical functions [18]. To comprehend how the intricate interactions between DEGs function, the PIN was developed. The biological significance of proteins was ascertained using topological centrality metrics with Network Analyse, a Cytoscape 3.9.1 plugin. Nodes in a network are frequently assessed using the three key metrics in network theory, such as the connection degree (k), BC, and closeness centrality (CC) value of nodes [19]. The number of nodes, linking elements at each node, network breadth, radius, density, number of neighbours at each node, clustering coefficient, and average shortest path length are further topological attributes [17].

### Interactomics analysis of hub gene

Hub genes are crucial components with the highest degree of interconnection and are crucial for comprehending the paths of biological networks. In order to examine the functional significance of the cellular map in identifying biomarkers and therapeutic targets, interactomics analysis portrays molecular interaction networks with physical links between neighbours [20]. We identified the top hub gene, close neighbourhood ranking network for addressing the gene’s novel function in the context of biological reactions using the Biological General Repository for Interaction Datasets (BioGRID) (BioGRID 4.4). The hub gene networks were selected using physical interactions and degree evidence ($\ge$ 70).

### Gene Ontology (GO) and molecular pathways analysis of DEGs

The primary bioinformatics method for integrating the characterisation of genes and gene products is GO analysis [21]. GO words fall into three categories: biological process, molecular function, and cellular component. Using taking into account statistically significant *P *> 0.05, DEGs for GO keywords were enhanced and examined by ShinyGO v0.741 [22]. EnrichR, a comprehensive gene set bioinformatics web tool, was used for pathway enrichment studies to investigate the common DEGs’ shared molecular signalling pathways. To find biological network pathways of DEGs in CAG, FG, and GC, we used pathway enrichment analyses from six databases, including KEGG [23], Rectome, Wiki, Panther, BioCarta, and BioPlanets. When choosing the top mentioned paths, we used the usual metric of *P* > 0.05.

### Recognition of transcriptional factors with connecting PPI network

Transcription factors (TFs) play a crucial part in a number of biological pathways by interacting in the vast protein complex network created by PPIs, which initiates and controls the transcription of genetic material [24]. We identified the main transcriptional factors using the hypergeometric *p*-value and the X2K web tool (regulatory networks platform) from the ChIP-seq experiments (ChEA) database [25]. Based on DEG signatures, the X2K online tool creates inferred TFs networks with connected PPI, producing upstream regulatory pathways. We discovered TFs by identifying proteins that physically interact with these transcription factors using the Genes2Networks (G2N) technique [26]. G2N is a powerful command-line and web-based programme that analyses genomic and proteomic data to interpret DEGs based on experimentally verified PPIs or protein complexes. With the use of this technology, researchers can filter TFs with links in protein network complexes to learn more about cell signalling cascades.

### Identification of protein kinase connecting with TFs and PPI

Phosphorylated targeted proteins are activated by protein kinases (PTKs), which are enzymes that dynamically control signalling proteins. PTKs were discovered using the kinase enrichment analysis (KEA) module of X2K. Mammalian protein DEG lists can be matched with the protein kinases predicted to phosphorylate them using the command-line tool KEA [27]. We also developed a regulatory kinase–substrate network that included PTKs, PPIs, and TFs with phosphorylation inside the extended subnetwork. The kinase–substrate network was developed using the human protein reference database (HPRD), PhosphoSite, phospho.ELM, NetworKIN, and Kinexus (www.kinexus.ca).

### Analysis of biological pathways in CAG, FA and GC

Biological pathway enrichment analysis of DEGs found in CAG, FA, and GC was performed using the FunRich tool (http://www.funrich.org) against the human FunRich background database [28].

#### Determination of mRNA expression levels of hub genes

Gene Expression Profiling Interactive Analysis (GEPIA) databases were used to analyse the mRNA expression levels of the hub genes in GC [29]. The GEPIA v1.0 does DEGs analysis, correlation analysis, patient survival analysis, similar gene recognition, and dimensionality reduction analysis based on the data from TCGA and GTEx. In this study, we utilised GEPIA to determine the expression of two hub genes with a threshold of *P* > 0.05 and a fold change of 2. An online tool called the Kaplan–Meier plotter [30] allows users to investigate the effect of 54,000 genes on survival in 21 different cancer types, including the largest datasets are for breast cancer (*n* = 6234), ovarian cancer (*n* = 2190), lung cancer (*n* = 3452), and gastrointestinal cancer (*n* = 1440). The major objective of the tool is to identify and validate survival biomarkers. Based on the GC database, a Kaplan–Meier Plotter online survival analysis of the key genes was performed. With 95% confidence intervals, the hazard ratio (HR) and log rank *P*-values were calculated.

#### Determination of the protein expression levels of the hub genes

The human protein atlas database (HPA v18.1) provides a wealth of transcriptome and proteome data from RNA-sequencing and immunohistochemistry research. The amount of each hub protein was assessed in this study using immunohistochemistry information from the HPA database.

## Results

### Screening and identification of DEGs

**Fig. 1:**
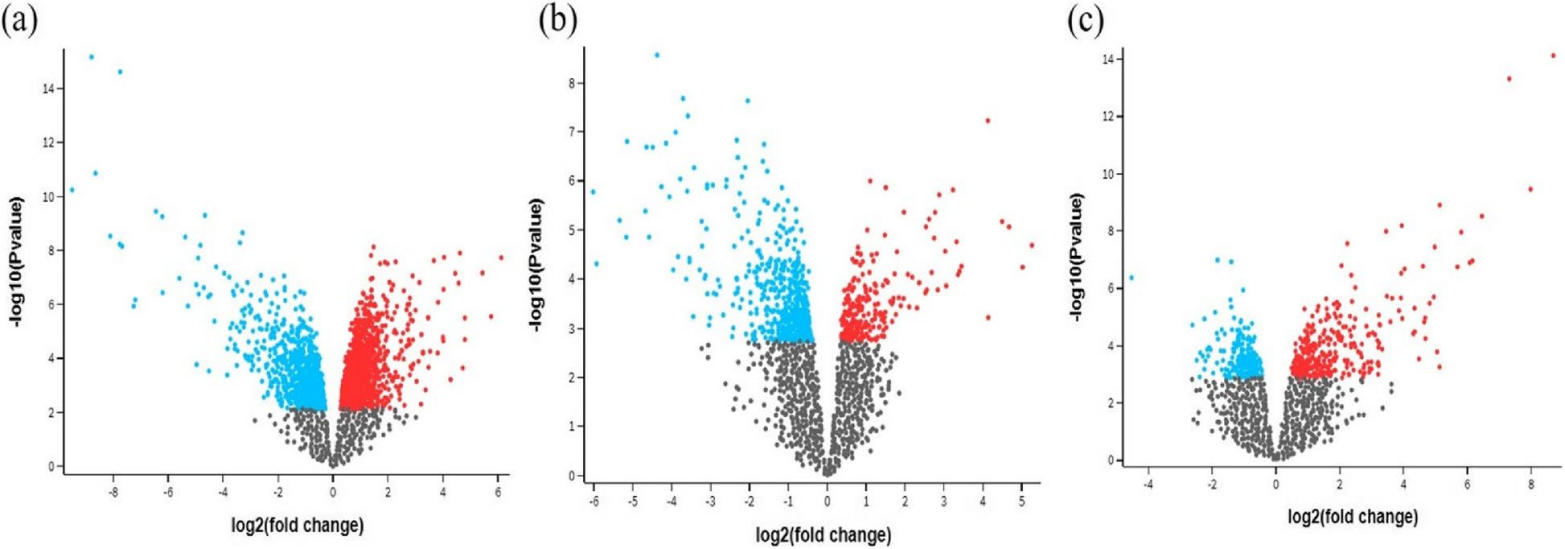
Volcano plot of all DEGs from **a** gastric cancer and follicular gastritis, **b** follicular gastritis and chronic atrophic gastritis, **c** chronic atrophic gastritis and gastric cancer, screening criteria: *P* < 0.05 and |log2FC|> 2. Up-regulated and down-regulated DEGs are indicated by red and blue, respectively. DEGs or differentially expressed genes, are a type of fold change analysis

Between CAG and GC, 92 DEGs were found, with 80 up-regulated and 12 down-regulated genes (Fig. 1A and Supplementary Table S1A). A total of 210 DEGs for FG and GC were found, including 121 up-regulated and 89 down-regulated genes (Fig. 1B and Supplementary Table S1B). In the meantime, 89 DEGs were found for CAG-FG, with 22 up-regulated and 67 down-regulated genes (Fig. 1C and Supplementary Table S1C).

### Identification of common DEGs

**Fig. 2:**
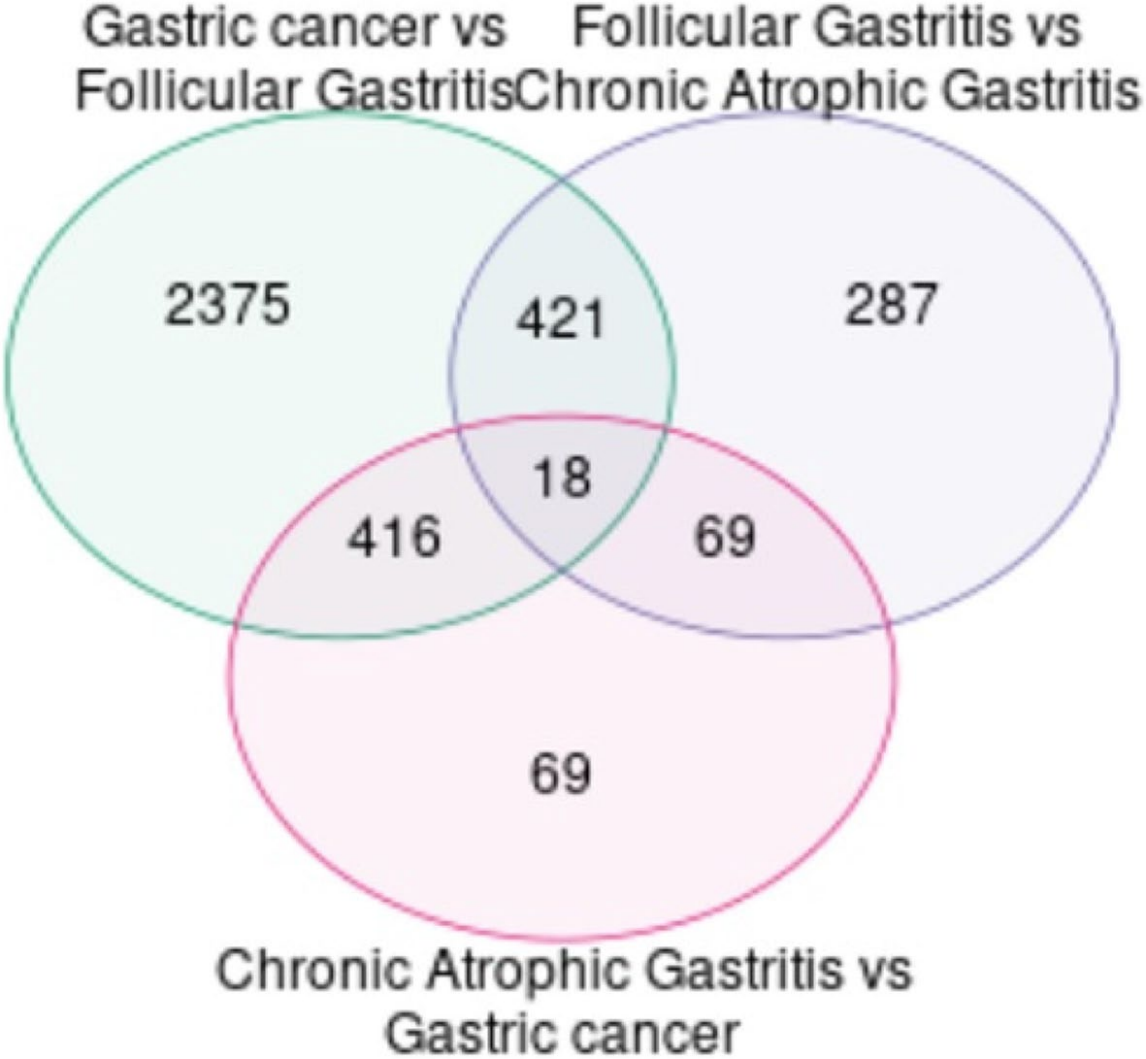
VENN diagram representing common genes among three groups, intersection of FG-CAG-GC

For this analysis, we used GEO2R tool of NCBI to find genes that are intersected among FG, CAG and GC. The intersection sets for FG, CAG, and GC are FG-CAG, GC-FG, and CAG-GC. The FG-CAG, GC-FG, and CAG-GC intersected genes are 421, 416, and 69, respectively. To determine the common genes among three groups, intersection of FG-CAG-GC was performed and a total of 17 gene is found in common, i.e., *CLDNI, CLDN4, NPNT, ABHD11, PLOD3, MCM7, TNFSF4, P4HB, CACNA1A, CIDEC, ENTPD3, DERL3, KCNE2, PGA4, PGA3, PGA5*, and *LIPF* (Fig. 2 and Supplementary Table S2).

### PIN construction

**Fig. 3:**
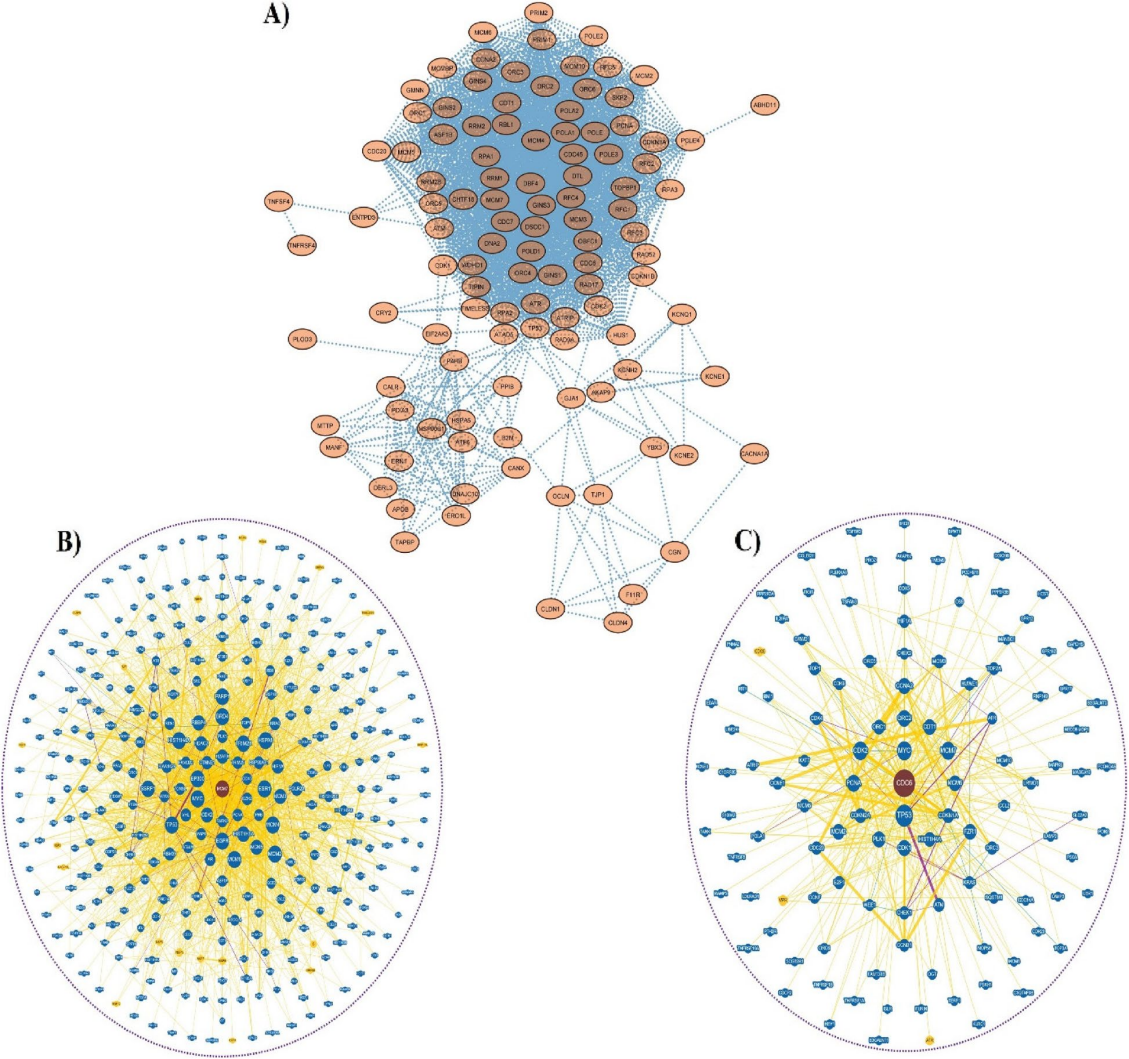
**A** Protein–protein interactions networks of the DEGs, interactome network analysis based on the physical interaction and degree evidence (≥ 70) of top two hub genes interaction using the Biological General Repository for Interaction Datasets (BioGRID): **B** MCM7 and **C** CDC6

We constructed the functional and physical network of the PPI between the DEGs of FG-CAG-GC by using the STRING database. To achieve the maximum number of interactions between DEGs and interacting functional partners, the network was further extended. The interaction score > 0.4 criteria was applied to a PPIs network of DEGs, and the minimum number of interactions was set to 50 in both shell1 and shell2, which led 110 nodes and 2103 edges (Fig. 3A). The two nodes that were chosen at random, the BC, degree, and the average clustering coefficient of the network nodes are all connected via the network’s shortest paths (Supplementary Table S3). A few closely coupled nodes made up the majority of the core network, it was discovered. Other nodes have a few characteristics that are common to the PPI network.

### Interactomics analysis of hub gene

Modern systems biology approaches that produce a rich context for protein function include interactomics as a key component. The interactome study only takes into account the expected physical network of PPIs with a score > 0.5. The amount of proteins interactions inside the PPI network was shown by the degree value of the hub genes, which was determined by the results of the topological analysis. We found the top 10 hub genes (*MCM7, CDC6, CDC45, MCM2, MCM4, CDK1, MCM3, CDK2, PCNA,* and *RFC4*) using the Network Analyst, which are highly nodes degree connections and reveal the therapeutic targets of GC. In order to address the novel role of the gene in the context of biological responses, we also carried out an investigation of interactomics-based interaction and degree evidence (*k* = 70) of top hub gene interactions with close neighbourhood proteins. Last but not least, we evaluated the interactome networks of hub genes like *MCM7* and *CDC6* (Fig. 3B and C), especially in relation to predictive biomarker for GC.

### Functional enrichment analysis of the DEGs

The biological functions of 114 genes were determined using the GO enrichment analyses that were performed for both up-regulated and down-regulated DEGs. The three distinct ontologies that the GO analysis is developing were annotated using the GO term database (biological process (BP), cellular component, and molecular function (MF)). GO enrichment analysis of up- and down-regulated DEGs across three categories at *P*-value threshold less than 0.5 is also shown, along with a selection of human species (BP, MF, and cellular component) (Supplementary Figure S1).

Following analysis of the GO enrichment results for the BP category, we revealed that DEGs were significantly enriched in the GO terms for nuclear cell cycle DNA replication, pre-replicative complex assembly, cell cycle DNA replication, double-strand break repair via break-induced replication, DNA strand elongation involved in DNA replication, DNA replication initiation, mitotic DNA replication, and DNA strand elongation (Supplementary Figure S1A). Additionally, the CC category contained enriched DEGs for the DNA replication preinitiation complex, CMG complex, alpha DNA polymerase:primase complex, GINS complex, replication fork protection complex, integrin alpha8-beta1 complex, DNA replication factor C complex, origin recognition complex, nuclear origin of replication recognition complex, and MCM complex (Supplementary Figure S1B). We have shown that procollagen-proline 4-dioxygenase activity, DNA replication origin binding, DNA clamp loading, protein-DNA loading ATPase activity, procollagen-proline dioxygenase activity, binding of the mismatch repair complex, single-stranded DNA helicase activity, DNA helicase activity, single-stranded DNA binding, catalytic activity, and acting on DNA were the main enriched DEGs for the MF category (Supplementary Figure S1C).

### Identification of crucial signalling pathways

**Fig. 4:**
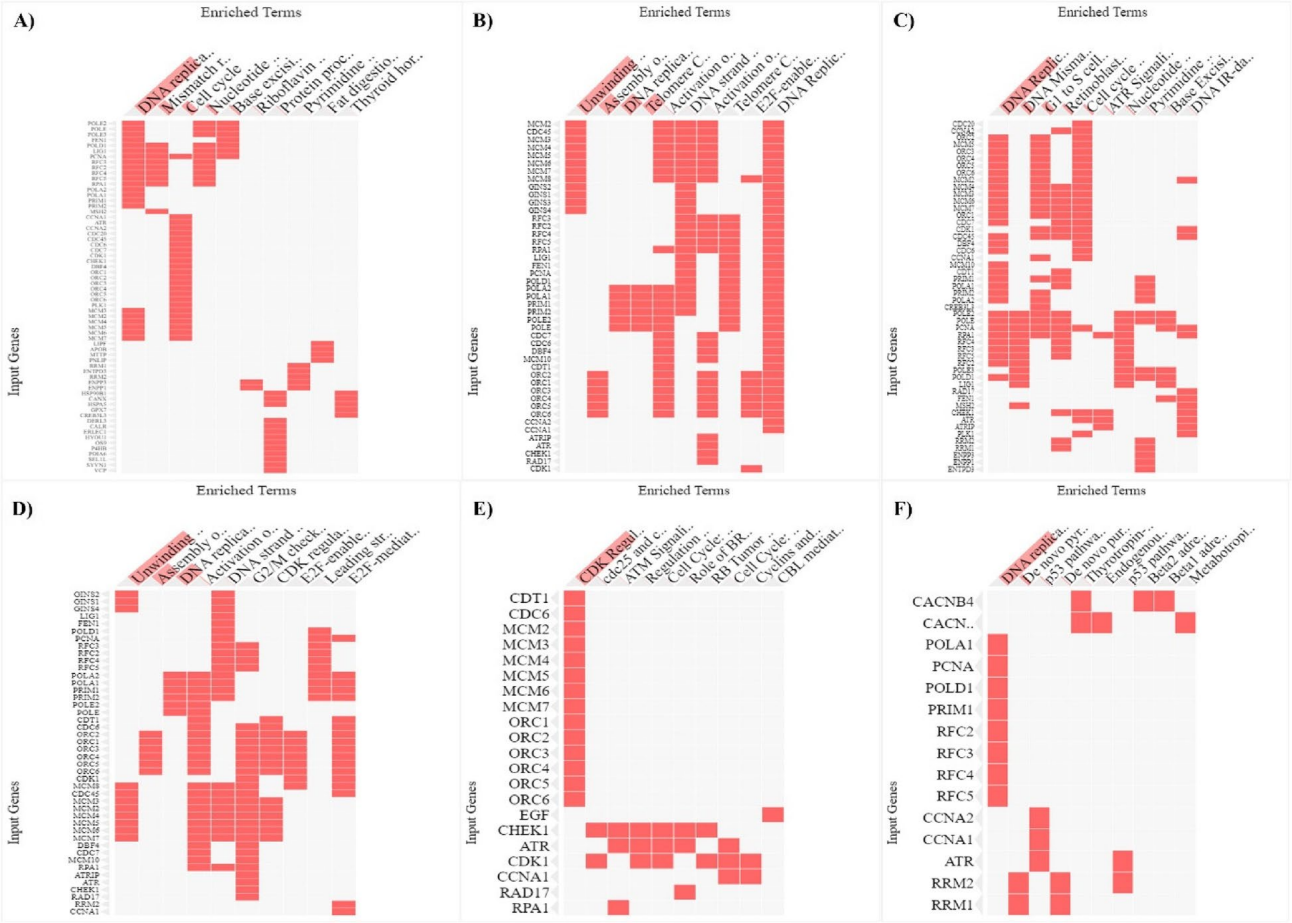
Functional enrichment of signalling pathways for the common DEGs in six pathway databases **A** KEGG, **B** Reactome, **C** Wikipathways, **D** BioPlanets, **E** BioCarta, and **F** Panther using a web-based bioinformatics programme EnrichR

One of the most important omics research techniques in the life sciences is pathways analysis, which aims to make sense of high-throughput biological data by identifying the biological signalling pathways involved in the genesis of complex disorders. KEGG, Reactome, Wiki, Panther, BioCarta, and BioPlanets are only a few of the six pathways databases used in gene set enrichment analysis, which was carried out using the web-based bioinformatics tool EnrichR. The top 10 signalling pathways based on the significance of *P* > 0.01 were taken into consideration when evaluating the pathways analysis associated to DEGs in FG-CAG-GC. All six databases share information on DNA replication regulation by CDK, ATM signalling, G1 to S cell control, nucleotide excision repair, activation of the pre-replication complex, and cell replication (Fig. 4A–F).

### Transcriptional regulatory networks analysis of DEGs related to FG-CAG-GC

The crucial molecules known as TFs directly maintain gene regulatory networks and control gene expression. The repression or activation of TFs, which are essential for many key cellular and biological processes and whose dysregulated TFs have been linked to the formation of neurological diseases, regulates the DEGs. With the help of the X2K online tool and the ChEA database, we identified the specific transcriptional factors impacting the expression of DEGs in FG-CAG-GC.

**Fig. 5:**
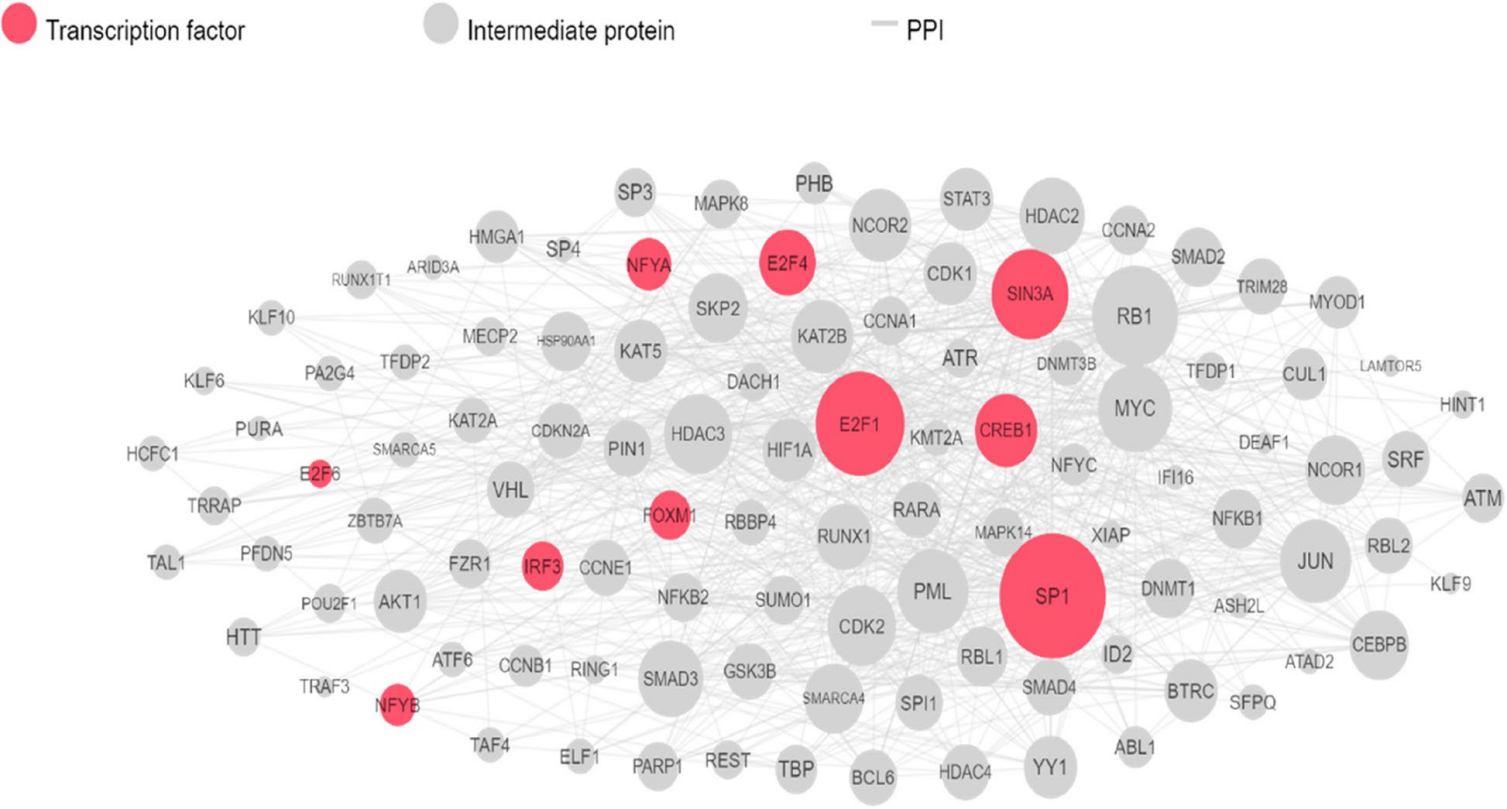
Transcription factor enrichment analysis with PPI network using Gene2Networks (G2N) algorithm. Pink nodes represent transcription factors and proteins connect with them in grey

Our TFs enrichment analysis (TFEA) selected the top 20 candidates of TFs based on the hypergeometric *p*-value, including E2F transcription factor 4 (p107/p130-binding) (ESF4), E2F transcription factor 1 (E2F1), E2F transcription factor 6 (E2F6), nuclear transcription factor Y, alpha (NFYA), nuclear transcription factor Y, beta (NFYB), SIN3 transcription regulator family member A (SIN3A), interferon regulatory factor 3 (IRF3), Forkhead boxes (FOXM1), zinc fingers (SP1), basic leucine zipper proteins (CREB1), basic leucine zipper proteins (FOS), homeoboxes (FBX3), RNA-binding motif containing (RBM) (NELFE), chromatin-modifying enzymes (KAT2A), basic leucine zipper proteins (ATF2), zinc fingers (SP2), zinc fingers, tripartite motif-containing (TRIM) (PML), basic leucine zipper proteins (CREB1), nuclear respiratory factor 1 (NRF1), and zinc fingers (AR) which could be shown altering gene function as CAG-GC disease progresses (Supplementary Figure S2). In order to evaluate the interactions between PPIs and TFs, we also employed the G2N method to find proteins that physically interact with these TFs. The regulatory network of linked TFs and the proteins that they interact with physically and functionally was shown based on the degree of the nodes (Fig. 5).

### Upstream regulatory pathway of kinase enrichment analysis

The kinase enrichment analysis result have shown that mitogen-activated protein kinase 14 (MAPK14), casein kinase 2, alpha 1 polypeptide (CSNK2A1), cyclin-dependent kinases (CDK1, CDK2, and CDK4), glycogen synthase kinase 3 beta (GSK3B), homeodomain interacting protein kinase 2 (HIPK2), mitogen-activated protein kinase 1 (MAPK1), ATM serine/threonine kinase (ATM), casein kinase 2 alpha 2 (CK2ALPHA), glycogen synthase kinase 3β (GSK3BETA), mitogen-activated protein kinase 8 (JNK1), protein kinase, DNA-activated, catalytic polypeptide (DNAPK), mitogen-activated protein kinase 3 (MAPK3), mitogen-activated protein kinase 3 (ERK1), V-akt murine thymoma viral oncogene homolog 1 (VKT1), protein kinase B alpha (PKBALPHA), DNA-dependent protein kinase subunit (PRKDC), and checkpoint kinase 1 (CHEK1) are the top protein kinases associated with FG-CAG-GC of intracellular signalling pathways (Supplementary Figure S3).

**Fig. 6:**
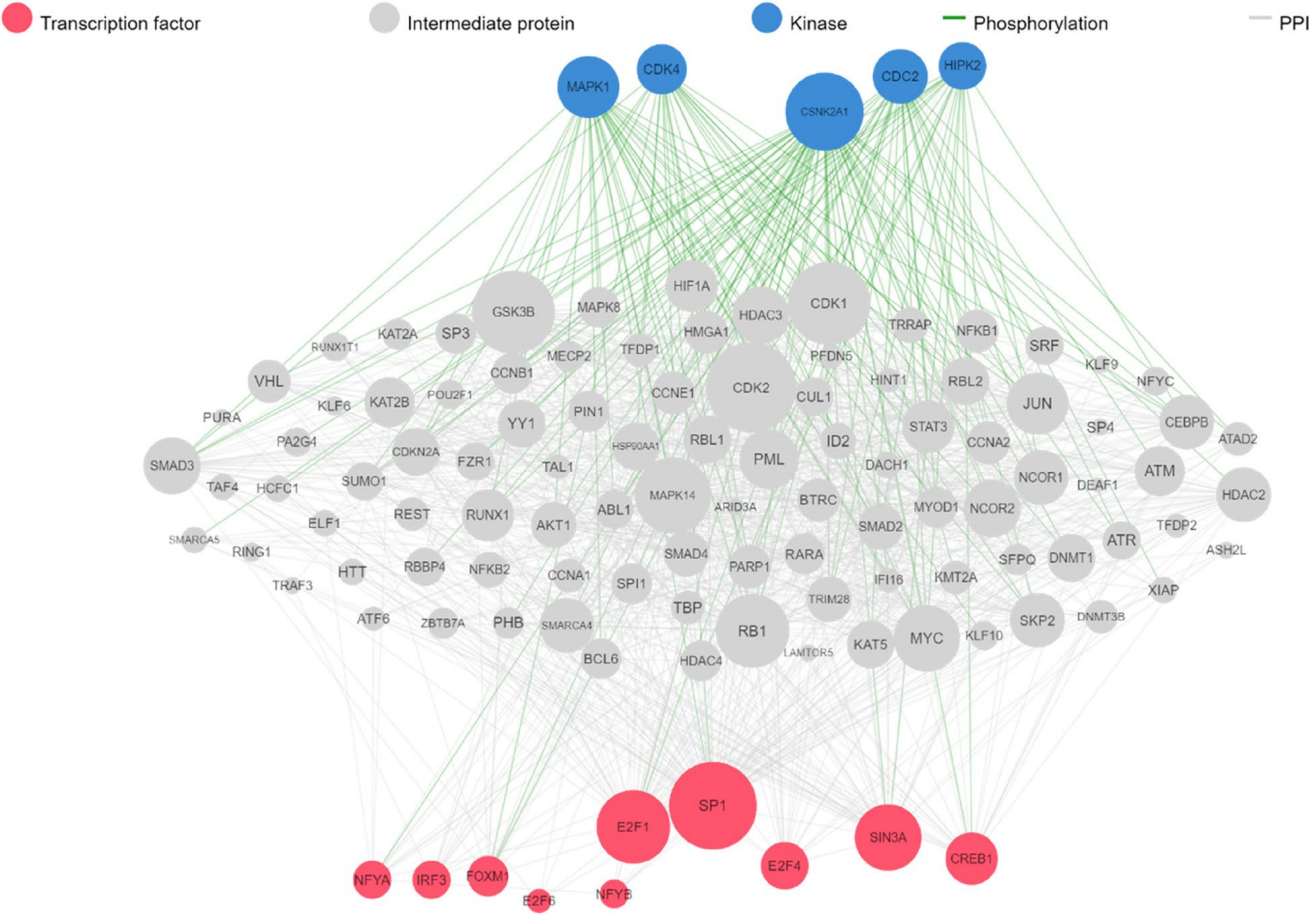
The enrichment analysis of kinase with transcription factors and PPI network. Red nodes represent the top transcription factors, blue nodes represent protein kinase, green network edges represent kinase-substrate phosphorylation interactions, grey edges represent physical protein–protein interactions network and red nodes show transcription factors

A kinase–substrate network, including PhosphoSite, phospho.ELM, NetworKIN, and Kinexus, was built using HPRD. The extended subnetwork of TFs and intermediate proteins was revealed by our bioinformatics research to have a regulatory kinase–substrate network that protein kinases activated phosphorylate substrates therein (Fig. 6).

### Determination of metabolic pathways in FG, CAG, and GC that DEGs share

The probable metabolic pathways associated with FG, CAG, and GC were investigated using the FunRich software. Our findings showed that the mitotic cell cycle (50%), DNA replication (46.51%), S-phase (40.70%), DNA synthesis (39.53%), mitotic G1-G1/S phase (34.88%), G1/S transition (34.88%), cell cycle checkpoints (33.72%), mitotic M-M/G1 phase (33.72%), G2/M checkpoints (32.56%), and activation of ATP in response to replication stress (31.40%) were the top 20 major biological pathways of FG-CAG (Supplementary Figure S4).

### mRNA expression levels of hub genes

**Fig. 7:**
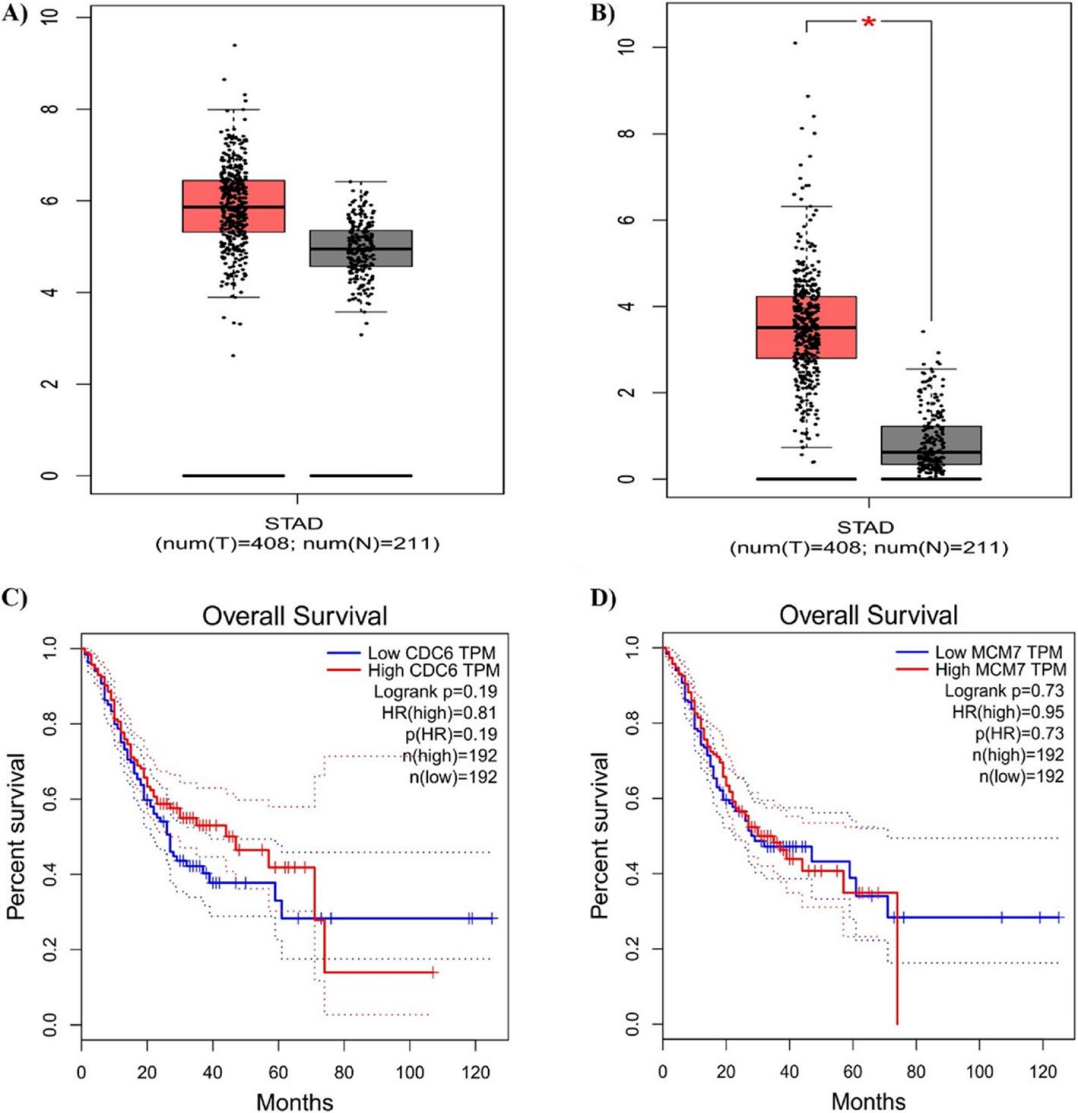
Significantly expressed genes in gastric cancer patients compared to healthy individuals. Red: tumour tissue; grey: normal tissues (*P* < 0.05), **A** MCM7 and **B** CDC6. Survival plot and prognostic information of the 2 hub genes. Red: high expression; black: low expression, **C** MCM7 and **D** CDC6

The two hub genes mRNA levels in tissue samples from GC and healthy individuals were compared using GEPIA. This showed that both genes were significantly expressed in GC specimens compared to usual stomach samples (*P* > 0.05, Fig. 7A–D).

### Hub protein expression in cancer tissues

**Fig. 8:**
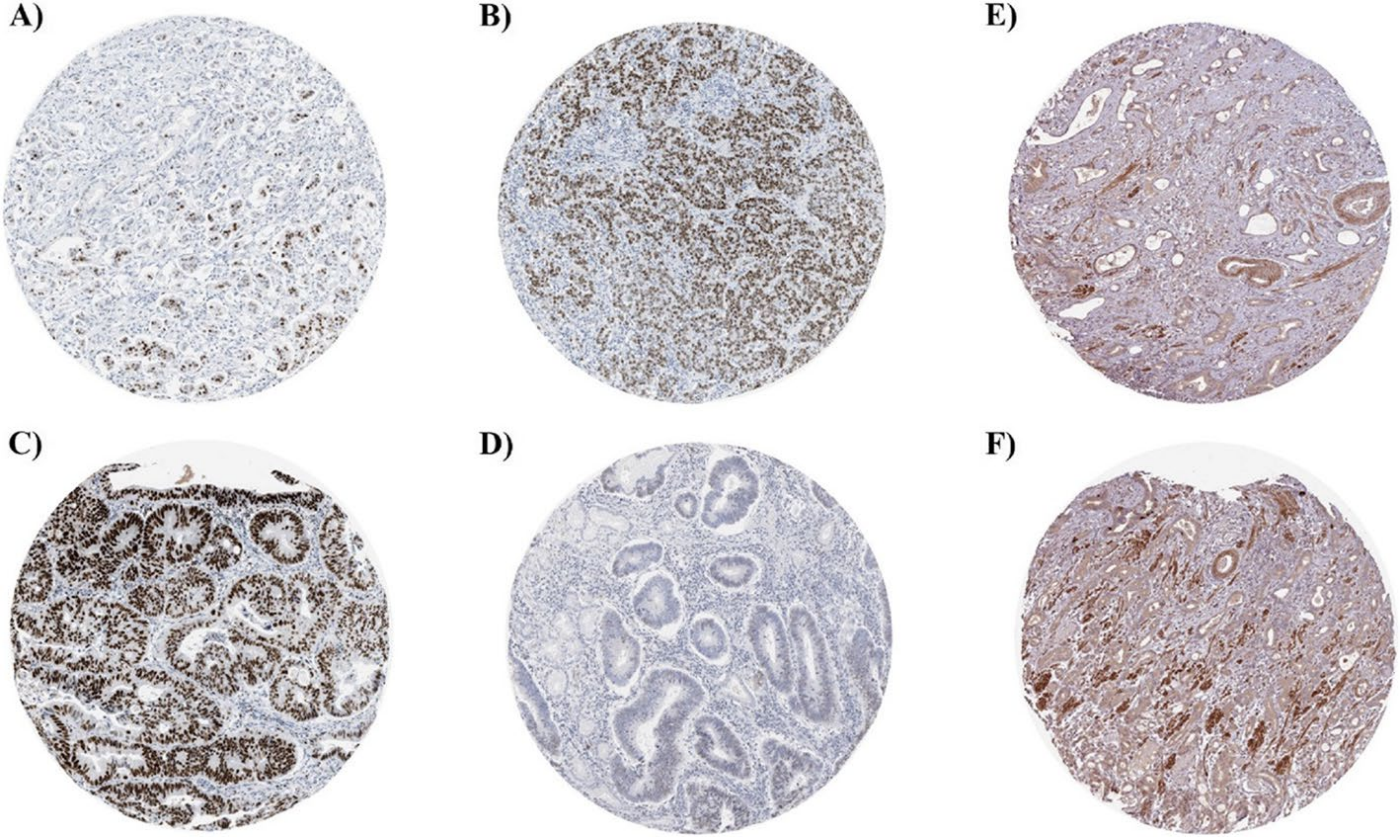
The hub protein expression in gastric cancer tissues. Images were taken from the Human Protein Atlas (http://www.proteinatlas.org) online database (HE, × 4). **A**, **B** MCM7 protein expression for stomach cancer of male patient (age 62, Patient ID: 2105) was high. **C**, **D** MCM7 protein expression for stomach cancer of male patient (age 59, Patient ID: 2378) was high. **E**, **F** CDC6 protein expression for stomach cancer of male patient (age 55, Patient ID: 3492) was moderate

**Fig. 9:**
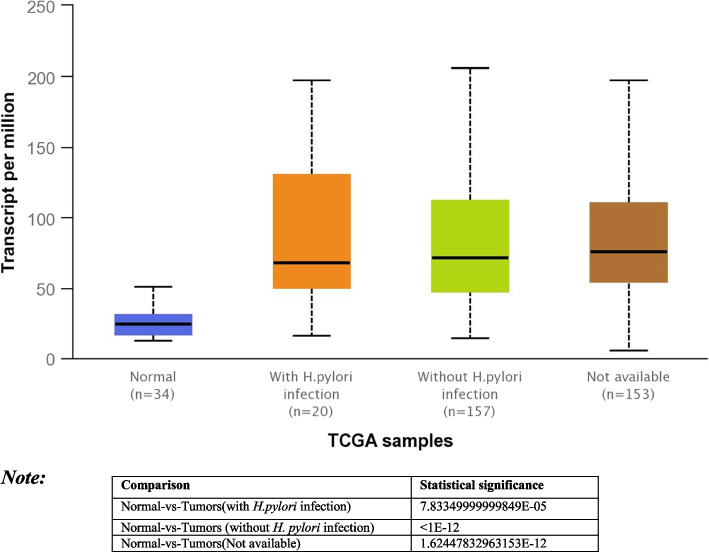
Quantification of *MCM7* expression in normal and tumour samples of stomach adenocarcinoma patients with or without *H. pylori* infection using UALCAN database (http://ualcan.path.uab.edu/analysis.html)

The Human Protein Atlas was used to analyse the two key DEGs’ protein expression in human GC tissue samples (Fig. 8). In contrast to the CDC6, which displayed moderate expression levels, the MCM7 protein displayed varied expression across GC and healthy gastric tissue samples (Fig. 8A, B, C, and Supplemental Figure S5A) (Fig. 8E, F and Supplementary Figure S5B). Further, the expression of *MCM7* is quantified in stomach adenocarcinoma (STDA) based on normal and tumour samples were compared with and without *H. pylori* infection (Fig. 9) using UALCAN database (http://ualcan.path.uab.edu/analysis.html) [31].

## Discussion

In recent years, chronic gastritis has gained a clinical focus despite being previously thought to be a common ageing occurrence and a non-pathological feature. Chronic gastritis is an immunopathological illness linked to *H. pylori* infection. CAG, a precursor stage of intestinal-type GC, develops due to the infection's persistence [32]. However, almost all GC patients experienced disease progression after treatment. The majority of GC cases are found to be in advanced stages, which leads in a relatively poor prognosis for survival. Identification of biomarkers or therapeutic targets is therefore crucial for enhancing GC diagnosis and prognosis [33]. Currently, it is understood that a long-term *H. pylori* infection is the basic cause of GC. Understanding how the molecular mechanisms of GC change in its early stages and finding potential biomarkers for the disease will help clinicians to make an early diagnosis, which will improve the prognosis for the patient. We employed a bioinformatics strategy to analyse microarray dataset in order to find useful prognostic indicators for FG-CAG-GC. We assessed the degree and main centralities, such as BC and CC, for each of the identified genes and important complexes (two clusters). In our analysis of the network and its subnetworks, the proteins MCM7 and CDC6 had the highest central indices (Figs. 3, 4 and 5). The application of networks or graph theory makes it possible to analyse various biological communication systems. PPI can be used to effectively understand and estimate the possibility of existing but unexplored connections between proteins/genes [34]. Many of the PINs have topological properties that are linked to protein essentiality. Its interconnectedness reveals the gene/relevance, proteins, and their topological roles, known as hubs, may be categorised depending on their location. A topological network analysis should theoretically disclose proteins that could be exploited as biomarkers or therapeutic targets, according to the theory. As a result, looking at these proteins could be a quick way to discover new GC genes and biomarkers [32]. Our research eventually led us to the conclusion that two genes (*MCM7* and *CDC6*), which were all enriched for the CDK regulation of DNA replication and mitotic M-M/G1 phase pathway, were associated with prognosis for GC.

MCM7 is one of the essential mini-chromosome maintenance proteins required for the beginning of genomic replication [35]. A crucial component of the pre-replication complex, which is involved in the formation of replication forks and the recruitment of additional proteins necessary for DNA replication, the MCM proteins form a hexameric protein complex [36]. Investigations have shown that the MCM4, 6, and 7 complexes serve as a DNA unwinding enzyme and have DNA helicase activity [37]. More and more details about MCM7’s function in the development of cancer are becoming available since it has been discovered to be amplified and overexpressed in a number of human malignancies [34]. The phosphorylation of MCM7 at Tyr-Y600 by EGFR, which promotes the proliferation of cancer cells, facilitates the creation and loading of the MCM complex [38]. E2F1 may be crucial in the development of gastric cancer by influencing the cell cycle pathway and modulating its target gene *MCM3*, which may interact with *MCM4*, *MCM5*, and *MCM7* [39]. In the 7q21–22 area of the GC chromosome, numerous genes, including *SHFM1*, *MCM7*, and *COL1A2*, have been identified as likely cancer candidate genes [40]. This amplicon contains two polycistrionic miRNA clusters, and the miR-106b-25 cluster, which is present in intron 13 of MCM7, was identified in the current investigation as being expressed in stomach tumours. The 7q21-22 amplification, MCM7, and its intronic miR-25 have also been conclusively demonstrated to represent the three primary molecular switches in the complex oncogenic circuits of gastric cancer [40]. Examined were the roles and mechanisms of MCM7 amplification and overexpression in the development of oesophageal cancer. ESCC cells multiplied, formed colonies, and migrated more readily as a result of MCM7’s stimulation of the AKT1/mTOR signalling pathway [41].

For the evaluation of GC and precancerous lesions, the combination of MCM7 and Ki67 may be more sensitive proliferation markers. We can do differential diagnosis in the pathological grade using MCM7 [42]. For GC patients, MCMs show potential diagnostic and prognostic values. GC tumours and metastatic lymph nodes had higher MCM2 expression levels than normal tissues. The prognosis is favourable for GC patients whose tumours do not exhibit MCM2. Since they are more accurate predictors of prognosis than conventional Ki-67 and PCNA, MCM2 and MCM5 are both beneficial prognostic indicators for GC patients. MCM2 helps to distinguish between gastric cardiac cancer and predicts stage III diffuse-type GC patients’ overall survival (OS) [43]. In individuals with diffuse-type GC, overexpressed MCM7 also indicates a low disease-specific survival rate. MCM7 knockdown reduces cell proliferation, colony formation, and invasion in AGS and NCI-N87 cells and is accompanied by an increase in apoptosis. In primary GC, gene amplification, somatic mutations, and mRNA upregulation are the key molecular mechanisms of MCMs [37].

One characteristic of the development of gastric tumours is the dysregulation of cell cycle components. Cell cycle progression is the outcome of cyclin-dependent kinase (CDK) activation. In GC, cyclin D1 and D2 expressions are up-regulated [44]. Additionally, in cocultured GC cells with an infection from *H. pylori*, cyclin D1 is up-regulated [45, 46]. Our pathway enrichment analysis revealed that the identified hub genes were significantly enriched in CDK regulation of DNA replication and mitotic M-M/G1 phase pathways. The mechanism of cellular proliferation produced by *H. pylori* infection is yet unknown, although *H. pylori* infection is also linked to increased cell proliferation of the host cells. In mammalian cells, the cell cycle, which controls the successive production and degradation of cyclins and cyclin-dependent kinases, regulates cellular proliferation. Cyclin D1 controls entry into the S phase and passing past the restriction point among other cyclins. Additionally, G1 phase lengthening and cellular proliferation rate are also accelerated by overexpressing cyclin D1 [46].

CDC6 (cell division cycle 6) is a cell cycle protein critical for the initiation of DNA replication. CDC6 functions as a checkpoint control that ensures DNA replication is finished before mitosis is started. It also functions as a regulator in the early stages of DNA replication. Many diseases (Meier-Gorlin Syndrome 5, Meier-Gorlin Syndrome 1) and various types of cancers were found to involve the dysregulation of CDC6 [47]. It is believed that CDC6 played a role in the emergence and progression of numerous malignancies. For instance, the expression of CDC6 was up-regulated in glioblastoma multiforme and strongly correlated with a bad prognostic profile [48]. It has been demonstrated that downregulating CDC6 prevents osteosarcoma carcinogenesis in both in vivo and in vitro [49]. Upregulated CDC6 expression was found in tumours, and reduction of CDC6 expression had a strong inhibitory effect on cancer formation and carcinogenesis [50]. CDC6 is connected to the loading of the MCM complex onto chromatin and is one of the most prevalent chromosomal replication licensors [51]. According to earlier studies, CDC6 is an essential part of the pre-replication complex that is involved in DNA replication in all eukaryotes [4, 52]. Because of CDC6’s crucial function in DNA replication, it was assumed that by controlling replication-related activities, it may affect transcription and proliferation [53]. Dysregulation of CDC6 can cause carcinogenesis and the emergence of various malignancies. When the expression of CDC6 was lowered, the proliferative ability would be severely constrained [47]. The current findings demonstrated that GC expressed *MCM7* at a higher level than normal stomach tissue and the potential of *MCM7* and *CDC6* as a biomarker for GC patients.

## Conclusion

The GEO dataset revealed that *MCM7* was related to the prognosis of GC. Bioinformatic analysis has revealed these genes to be effective and trustworthy molecular indicators for the diagnosis and prognosis of GC, revealing a new and promising treatment target for the disease. Furthermore, pathway enrichment analysis demonstrated that these genes are important for the CDK regulation of DNA replication and the mitotic M-M/G1 phase pathway. It is crucial to acknowledge the research’s limitations, such as the fact that the crucial roles of these hub genes in the GC were only hypothetically inferred using public information. Additional experimental research is required to support the findings of the current study.

### Supplementary Information

**Additional file 1.** Supplementary materials.

## Data Availability

All the data we generated in this paper is available in the body of the manuscript, supporting tables, and figures. We do not have any ethical or legal considerations for not making our data publicly available.

## Abbreviations

GC: Gastric cancer
DEGs: Differentially expressed genes
KEGG: Kyoto Encyclopedia of Genes and Genomes
PPI: Protein–protein interaction
CDK: Cyclin-dependent kinase
GEPIA: Gene Expression Profiling Interactive Analysis
CAG: Chronic atrophic gastritis
*H. pylori*: *Helicobacter pylori*
GEO: Gene Expression Omnibus
STRING: Search Tool for the Retrieval of Interacting Genes
PIN: Protein interaction network
BioGRID: Biological General Repository for Interaction Datasets
GO: Gene Ontology
TF: Transcription factors
HPRD: Human Protein Reference Database
NCBI: National Center for Biotechnology Information
