# Supplementary material for: Early diagnostic and prognostic biomarkers for gastric cancer: systems-level molecular basis of subsequent alterations in gastric mucosa from chronic atrophic gastritis to gastric cancer
Source: J Genet Eng Biotechnol. 2023 Aug 18;21:86. doi: 10.1186/s43141-023-00539-0 (PMC10439097; doi:10.1186/s43141-023-00539-0)

**Early Diagnostic and Prognostic Biomarkers for Gastric cancer: Systems-level molecular basis of subsequent alterations in gastric mucosa from chronic atrophic gastritis to gastric cancer**

**Supplementary Materials**

**Supplementary Table S1A.** List of DEGs extracted from GEO datasets of CAG and GC

| Probe  ID | Log FC | Ave Expr | T-test | P-Value | Adj P-Val | B | ENTREZ ID | Gene Name | Gene Symbol |
| --- | --- | --- | --- | --- | --- | --- | --- | --- | --- |
| 8052753 | 8.697242 | 11.24582 | 35.95921 | 1.05E-14 | 3.25E-10 | 16.73829 | 200504 | gastrokine 2 | GKN2 |
| 8042431 | 7.985035 | 10.41886 | 15.97108 | 4.24E-10 | 4.36E-06 | 11.93145 | 56287 | gastrokine 1 | GKN1 |
| 8126324 | 7.315712 | 12.26181 | 31.22845 | 6.81E-14 | 1.05E-09 | 16.19085 | 5225 | progastricsin | PGC |
| 8169263 | 6.458674 | 11.56312 | 13.47231 | 3.64E-09 | 2.25E-05 | 10.43333 | 340547 | V-set and immunoglobulin domain containing 1 | VSIG1 |
| 7928982 | 6.162588 | 10.17233 | 10.08031 | 1.27E-07 | 2.75E-04 | 7.630131 | 8513 | lipase F, gastric type | LIPF |
| 8118061 | 6.077906 | 10.70654 | 9.954504 | 1.48E-07 | 2.84E-04 | 7.504336 | 135656 | mucin like 3 | MUCL3 |
| 8070574 | 5.810443 | 11.13351 | 12.15757 | 1.31E-08 | 5.04E-05 | 9.469373 | 7032 | trefoil factor 2 | TFF2 |
| 8179221 | 5.691341 | 10.61759 | 9.675861 | 2.07E-07 | 3.03E-04 | 7.219242 | 135656 | mucin like 3 | MUCL3 |
| 7945595 | 5.140484 | 10.97946 | 14.44591 | 1.52E-09 | 1.17E-05 | 11.06421 | 4588 | mucin 6, oligomeric mucus/gel-forming | MUC6 |
| 7927529 | 5.13431 | 8.761934 | 4.479959 | 5.78E-04 | 0.044237 | -0.07422 | 4477 | microseminoprotein beta | MSMB |
| 8121936 | 5.054199 | 8.631479 | 5.13638 | 1.75E-04 | 0.025614 | 1.070654 | 352999 | chromosome 6 open reading frame 58 | C6orf58 |
| 8070579 | 4.976677 | 12.25139 | 11.0389 | 4.26E-08 | 1.31E-04 | 8.532091 | 7031 | trefoil factor 1 | TFF1 |
| 8081826 | 4.95053 | 8.04807 | 7.880644 | 2.19E-06 | 0.00225 | 5.148477 | 7348 | uroplakin 1B | UPK1B |
| 8082928 | 4.819431 | 11.93981 | 7.522371 | 3.66E-06 | 0.003054 | 4.68214 | 51208 | claudin 18 | CLDN18 |
| 8061894 | 4.691287 | 9.053731 | 5.751376 | 5.98E-05 | 0.01473 | 2.087089 | 92747 | BPI fold containing family B member 1 | BPIFB1 |
| 8098246 | 4.683094 | 9.852317 | 6.750327 | 1.18E-05 | 0.005942 | 3.611418 | 11199 | annexin A10 | ANXA10 |
| 8037197 | 4.610868 | 10.97497 | 9.719013 | 1.96E-07 | 3.03E-04 | 7.263986 | 284340 | C-X-C motif chemokine ligand 17 | CXCL17 |
| 8069582 | 4.484895 | 6.091056 | 4.824928 | 3.06E-04 | 0.033931 | 0.534583 | 5651 | transmembrane serine protease 15 | TMPRSS15 |
| 8090955 | 4.347819 | 8.227204 | 7.258378 | 5.41E-06 | 0.003973 | 4.326295 | 51146 | alpha-1,4-N-acetylglucosaminyltransferase | A4GNT |
| 8148220 | 4.035268 | 9.475485 | 9.532069 | 2.47E-07 | 3.46E-04 | 7.068548 | 654463 | fer-1 like family member 6 | FER1L6 |
| 8148548 | 3.961305 | 11.47686 | 6.726213 | 1.22E-05 | 0.006071 | 3.576486 | 8000 | prostate stem cell antigen | PSCA |
| 8044212 | 3.944693 | 10.78908 | 12.6576 | 7.93E-09 | 4.08E-05 | 9.852329 | 6819 | sulfotransferase family 1C member 2 | SULT1C2 |
| 7999920 | 3.92555 | 8.202067 | 9.256908 | 3.48E-07 | 4.66E-04 | 6.773205 | 2813 | glycoprotein 2 | GP2 |
| 8044067 | 3.917183 | 9.158208 | 7.822084 | 2.38E-06 | 0.002358 | 5.073539 | 389015 | solute carrier family 9 member A4 | SLC9A4 |
| 7909946 | 3.860784 | 8.344184 | 7.126871 | 6.59E-06 | 0.004326 | 4.145081 | 400823 | family with sequence similarity 177 member B | FAM177B |
| 8136336 | 3.647232 | 10.5839 | 7.802356 | 2.45E-06 | 0.002358 | 5.048181 | 57016 | aldo-keto reductase family 1 member B10 | AKR1B10 |
| 7954589 | 3.589176 | 7.478453 | 6.559039 | 1.59E-05 | 0.007115 | 3.3318 | 387849 | RAB15 effector protein | REP15 |
| 8147132 | 3.479994 | 11.6995 | 7.915844 | 2.08E-06 | 0.002215 | 5.193282 | 760 | carbonic anhydrase 2 | CA2 |
| 8112668 | 3.454754 | 8.467595 | 12.19576 | 1.26E-08 | 5.04E-05 | 9.499367 | 51301 | glucosaminyl (N-acetyl) transferase 4 | GCNT4 |
| 8046824 | 3.33013 | 7.985639 | 5.255792 | 1.41E-04 | 0.022559 | 1.272496 | 401024 | fibrous sheath interacting protein 2 | FSIP2 |
| 8000799 | 3.270265 | 9.326659 | 6.395904 | 2.06E-05 | 0.008315 | 3.088755 | 79153 | glycerophosphodiester phosphodiesterase domain containing 3 | GDPD3 |
| 7910466 | 3.250207 | 8.884672 | 6.422634 | 1.97E-05 | 0.00822 | 3.128869 | 10753 | calpain 9 | CAPN9 |
| 8083415 | 3.239703 | 7.787005 | 5.532675 | 8.70E-05 | 0.017773 | 1.732277 | 13 | arylacetamide deacetylase | AADAC |
| 8138363 | 3.227716 | 8.926489 | 6.890219 | 9.46E-06 | 0.005473 | 3.812262 | 25928 | sclerostin domain containing 1 | SOSTDC1 |
| 8091799 | 3.224658 | 8.384558 | 4.537243 | 5.20E-04 | 0.042169 | 0.027888 | 165679 | serine palmitoyltransferase small subunit B | SPTSSB |
| 8050619 | 3.214858 | 6.545053 | 5.335345 | 1.23E-04 | 0.02091 | 1.405789 | 338 | apolipoprotein B | APOB |
| 8149142 | 3.20619 | 6.796031 | 4.669166 | 4.07E-04 | 0.037362 | 0.26154 | 1670 | defensin alpha 5 | DEFA5 |
| 8121152 | 3.191932 | 8.007201 | 4.612551 | 4.52E-04 | 0.039322 | 0.16153 | 10690 | fucosyltransferase 9 | FUT9 |
| 8110971 | 3.186848 | 9.582282 | 5.64918 | 7.12E-05 | 0.015802 | 1.922221 | 134147 | carboxymethylenebutenolidase homolog | CMBL |
| 7990555 | 3.110189 | 7.911842 | 5.923321 | 4.48E-05 | 0.012327 | 2.360768 | 145957 | neuregulin 4 | NRG4 |
| 8020827 | 3.035035 | 6.121752 | 6.009308 | 3.88E-05 | 0.011841 | 2.495874 | 4225 | meprin A subunit beta | MEP1B |
| 8093950 | 3.000166 | 10.88457 | 4.39765 | 6.74E-04 | 0.046831 | -0.22158 | 6286 | S100 calcium binding protein P | S100P |
| 7946946 | 2.995013 | 7.155515 | 4.686665 | 3.94E-04 | 0.037342 | 0.292372 | 7166 | tryptophan hydroxylase 1 | TPH1 |
| 8099255 | 2.936161 | 8.582306 | 4.423063 | 6.43E-04 | 0.045578 | -0.176 | 768239 | prosaposin like 1 | PSAPL1 |
| 8045664 | 2.915482 | 9.420512 | 5.947454 | 4.30E-05 | 0.012159 | 2.398805 | 130576 | LY6/PLAUR domain containing 6B | LYPD6B |
| 8103769 | 2.896624 | 10.7994 | 5.897051 | 4.68E-05 | 0.012768 | 2.319257 | 3248 | 15-hydroxyprostaglandin dehydrogenase | HPGD |
| 8096580 | 2.887756 | 6.533268 | 4.672691 | 4.04E-04 | 0.037342 | 0.267753 | 4547 | microsomal triglyceride transfer protein | MTTP |
| 8035648 | 2.838062 | 8.940146 | 6.334331 | 2.28E-05 | 0.008867 | 2.995921 | 53345 | transmembrane 6 superfamily member 2 | TM6SF2 |
| 8091071 | 2.825893 | 7.220167 | 7.203509 | 5.88E-06 | 0.004017 | 4.251008 | 5948 | retinol binding protein 2 | RBP2 |
| 7923850 | 2.771563 | 8.254911 | 4.567217 | 4.92E-04 | 0.041076 | 0.081161 | 115019 | solute carrier family 26 member 9 | SLC26A9 |
| 8043981 | 2.751601 | 9.243472 | 4.434182 | 6.30E-04 | 0.04503 | -0.15609 | 7850 | interleukin 1 receptor type 2 | IL1R2 |
| 7955297 | 2.695201 | 8.702345 | 5.482472 | 9.49E-05 | 0.01842 | 1.649781 | 362 | aquaporin 5 | AQP5 |
| 8092682 | 2.690688 | 9.931717 | 4.901023 | 2.66E-04 | 0.032348 | 0.666803 | 6750 | somatostatin | SST |
| 8100853 | 2.575832 | 7.218258 | 5.143098 | 1.72E-04 | 0.025478 | 1.082064 | 2638 | GC vitamin D binding protein | GC |
| 8121649 | 2.490081 | 6.141384 | 5.352079 | 1.19E-04 | 0.020764 | 1.433706 | 222546 | regulatory factor X6 | RFX6 |
| 8016523 | 2.489047 | 6.462642 | 8.387838 | 1.08E-06 | 0.001238 | 5.77709 | 2695 | gastric inhibitory polypeptide | GIP |
| 8078971 | 2.4859 | 7.049659 | 6.69614 | 1.28E-05 | 0.006114 | 3.532796 | 956 | ectonucleoside triphosphate diphosphohydrolase 3 | ENTPD3 |
| 8171449 | 2.450295 | 7.661203 | 5.513884 | 8.99E-05 | 0.018014 | 1.701444 | 59272 | angiotensin converting enzyme 2 | ACE2 |
| 7929466 | 2.447178 | 10.82044 | 6.284074 | 2.47E-05 | 0.009066 | 2.919703 | 1562 | cytochrome P450 family 2 subfamily C member 18 | CYP2C18 |
| 8044204 | 2.439289 | 7.152012 | 4.345805 | 7.43E-04 | 0.049718 | -0.31479 | 442038 | sulfotransferase family 1C member 3 | SULT1C3 |
| 7918794 | 2.387066 | 7.074798 | 7.605029 | 3.25E-06 | 0.002863 | 4.791409 | 270 | adenosine monophosphate deaminase 1 | AMPD1 |
| 8101893 | 2.371238 | 10.84161 | 4.597862 | 4.64E-04 | 0.039779 | 0.135516 | 126 | alcohol dehydrogenase 1C (class I), gamma polypeptide | ADH1C |
| 7920642 | 2.364119 | 11.77227 | 9.107495 | 4.20E-07 | 5.18E-04 | 6.608908 | 4582 | mucin 1, cell surface associated | MUC1 |
| 7999909 | 2.358316 | 10.37709 | 5.340257 | 1.22E-04 | 0.020877 | 1.413988 | 51704 | G protein-coupled receptor class C group 5 member B | GPRC5B |
| 8066493 | 2.356636 | 10.06945 | 5.861758 | 4.97E-05 | 0.013264 | 2.263318 | 6590 | secretory leukocyte peptidase inhibitor | SLPI |
| 7976322 | 2.352343 | 8.207992 | 5.482469 | 9.49E-05 | 0.01842 | 1.649776 | 1113 | chromogranin A | CHGA |
| 8067125 | 2.324893 | 10.65256 | 6.817609 | 1.06E-05 | 0.005727 | 3.708397 | 8537 | brain enriched myelin associated protein 1 | BCAS1 |
| 7972461 | 2.321694 | 6.623627 | 6.124315 | 3.21E-05 | 0.010771 | 2.674737 | 6564 | solute carrier family 15 member 1 | SLC15A1 |
| 8101429 | 2.248967 | 11.15781 | 4.754245 | 3.48E-04 | 0.035565 | 0.41107 | 51316 | placenta associated 8 | PLAC8 |
| 8171624 | 2.229341 | 7.228373 | 4.61991 | 4.46E-04 | 0.03928 | 0.174551 | 10149 | adhesion G protein-coupled receptor G2 | ADGRG2 |
| 8046833 | 2.225706 | 6.197316 | 4.454855 | 6.06E-04 | 0.044588 | -0.11908 | 401024 | fibrous sheath interacting protein 2 | FSIP2 |
| 8126820 | 2.193614 | 6.862371 | 6.300113 | 2.41E-05 | 0.009049 | 2.944071 | 266977 | adhesion G protein-coupled receptor F1 | ADGRF1 |
| 8046124 | 2.170334 | 6.505141 | 5.42935 | 1.04E-04 | 0.018887 | 1.562065 | 10170 | dehydrogenase/reductase 9 | DHRS9 |
| 8029136 | 2.156187 | 9.811041 | 4.754304 | 3.48E-04 | 0.035565 | 0.411174 | 973 | CD79a molecule | CD79A |
| 8060805 | 2.110497 | 6.9539 | 6.290694 | 2.44E-05 | 0.009066 | 2.929766 | 1114 | chromogranin B | CHGB |
| 7980080 | 2.076789 | 9.081937 | 5.788997 | 5.61E-05 | 0.014425 | 2.147368 | 957 | ectonucleoside triphosphate diphosphohydrolase 5 (inactive) | ENTPD5 |
| 7916112 | 2.058002 | 6.380018 | 9.698729 | 2.01E-07 | 3.03E-04 | 7.242981 | 5865 | RAB3B, member RAS oncogene family | RAB3B |
| 8162492 | 2.052526 | 7.2081 | 5.649128 | 7.12E-05 | 0.015802 | 1.922136 | 8789 | fructose-bisphosphatase 2 | FBP2 |
| 7989023 | 2.028465 | 9.327444 | 6.836157 | 1.03E-05 | 0.005727 | 3.735008 | 5873 | RAB27A, member RAS oncogene family | RAB27A |
| 8013660 | 2.007309 | 8.273509 | 5.747465 | 6.02E-05 | 0.01473 | 2.080809 | 230 | aldolase, fructose-bisphosphate C | ALDOC |
| 7953200 | -2.04773 | 8.983405 | -5.22639 | 1.49E-04 | 0.023281 | 1.222994 | 894 | cyclin D2 | CCND2 |
| 8161884 | -2.09714 | 6.729009 | -5.32332 | 1.25E-04 | 0.021232 | 1.385707 | 158471 | prune homolog 2 with BCH domain | PRUNE2 |
| 8095680 | -2.1265 | 7.349535 | -4.94718 | 2.45E-04 | 0.030975 | 0.746612 | 3576 | C-X-C motif chemokine ligand 8 | CXCL8 |
| 8131803 | -2.15289 | 5.658059 | -5.22898 | 1.48E-04 | 0.023281 | 1.227362 | 3569 | interleukin 6 | IL6 |
| 7982597 | -2.20782 | 9.248025 | -4.73263 | 3.62E-04 | 0.035813 | 0.373178 | 7057 | thrombospondin 1 | THBS1 |
| 8096301 | -2.23996 | 6.932031 | -6.69153 | 1.29E-05 | 0.006114 | 3.526087 | 6696 | secreted phosphoprotein 1 | SPP1 |
| 8170648 | -2.24171 | 9.735088 | -4.38001 | 6.97E-04 | 0.047867 | -0.25327 | 633 | biglycan | BGN |
| 8116921 | -2.29121 | 7.744802 | -4.9418 | 2.48E-04 | 0.03115 | 0.737329 | 1906 | endothelin 1 | EDN1 |
| 8139207 | -2.36903 | 7.290131 | -5.08672 | 1.91E-04 | 0.026975 | 0.986103 | 3624 | inhibin subunit beta A | INHBA |
| 8038725 | -2.48177 | 7.955342 | -4.75875 | 3.45E-04 | 0.035565 | 0.418957 | 5655 | kallikrein related peptidase 10 | KLK10 |
| 8092726 | -2.6192 | 8.318253 | -6.3914 | 2.08E-05 | 0.008315 | 3.081982 | 9076 | claudin 1 | CLDN1 |
| 8065412 | -4.52636 | 6.638361 | -8.98948 | 4.89E-07 | 5.80E-04 | 6.477145 | 1469 | cystatin SN | CST1 |

**Supplementary Table S1B.** List of DEGs extracted from GEO datasets of FG and GC

| Probe ID | Log FC | Ave Expr | t-test | P- Value | Adj P-Val | B | ENTREZ ID | Gene Name | Gene Symbol |
| --- | --- | --- | --- | --- | --- | --- | --- | --- | --- |
| 7928982 | 9.488561 | 10.17233 | 18.36433 | 7.07E-11 | 5.45E-07 | 14.33479 | 8513 | lipase F, gastric type | LIPF |
| 8052753 | 8.784171 | 11.24582 | 42.97278 | 9.90E-16 | 3.05E-11 | 20.29932 | 200504 | gastrokine 2 | GKN2 |
| 8042431 | 8.645297 | 10.41886 | 20.4598 | 1.74E-11 | 1.79E-07 | 15.34297 | 56287 | gastrokine 1 | GKN1 |
| 8126324 | 7.739608 | 12.26181 | 39.09101 | 3.48E-15 | 5.36E-11 | 19.86656 | 5225 | progastricsin | PGC |
| 7948433 | 7.252419 | 10.04833 | 8.262112 | 1.29E-06 | 3.97E-04 | 5.775006 | 2694 | cobalamin binding intrinsic factor | CBLIF |
| 7972923 | 7.194158 | 10.14352 | 8.664125 | 7.48E-07 | 2.75E-04 | 6.295169 | 496 | ATPase H+/K+ transporting subunit beta | ATP4B |
| 8169263 | 6.446389 | 11.56312 | 15.91033 | 4.45E-10 | 2.75E-06 | 12.90953 | 340547 | V-set and immunoglobulin domain containing 1 | VSIG1 |
| 8070574 | 6.210781 | 11.13351 | 15.37615 | 6.88E-10 | 3.03E-06 | 12.55771 | 7032 | trefoil factor 2 | TFF2 |
| 8036110 | 6.202385 | 10.22334 | 9.127824 | 4.10E-07 | 2.05E-04 | 6.86974 | 495 | ATPase H+/K+ transporting subunit alpha | ATP4A |
| 8068369 | 5.58718 | 8.83974 | 10.08735 | 1.26E-07 | 9.72E-05 | 7.977812 | 9992 | potassium voltage-gated channel subfamily E regulatory subunit 2 | KCNE2 |
| 8037197 | 5.379158 | 10.97497 | 13.41584 | 3.84E-09 | 1.18E-05 | 11.11449 | 284340 | C-X-C motif chemokine ligand 17 | CXCL17 |
| 8136187 | 5.278793 | 8.614565 | 8.270458 | 1.27E-06 | 3.97E-04 | 5.786019 | 1358 | carboxypeptidase A2 | CPA2 |
| 8118061 | 4.977619 | 10.70654 | 9.646089 | 2.14E-07 | 1.38E-04 | 7.481288 | 135656 | mucin like 3 | MUCL3 |
| 7927529 | 4.961601 | 8.761934 | 5.122451 | 1.79E-04 | 0.006568 | 0.92858 | 4477 | microseminoprotein beta | MSMB |
| 8044067 | 4.912353 | 9.158208 | 11.60653 | 2.31E-08 | 3.56E-05 | 9.532736 | 389015 | solute carrier family 9 member A4 | SLC9A4 |
| 8082928 | 4.89 | 11.93981 | 9.030916 | 4.64E-07 | 2.17E-04 | 6.75185 | 51208 | claudin 18 | CLDN18 |
| 8070579 | 4.829271 | 12.25139 | 12.67453 | 7.80E-09 | 1.85E-05 | 10.49845 | 7031 | trefoil factor 1 | TFF1 |
| 8148548 | 4.70224 | 11.47686 | 9.447157 | 2.74E-07 | 1.66E-04 | 7.250264 | 8000 | prostate stem cell antigen | PSCA |
| 7945595 | 4.659015 | 10.97946 | 15.49169 | 6.25E-10 | 3.03E-06 | 12.6352 | 4588 | mucin 6, oligomeric mucus/gel-forming | MUC6 |
| 7923850 | 4.540428 | 8.254911 | 8.852944 | 5.84E-07 | 2.40E-04 | 6.532352 | 115019 | solute carrier family 26 member 9 | SLC26A9 |
| 7903945 | 4.511354 | 7.771694 | 4.818055 | 3.10E-04 | 0.009178 | 0.383396 | 27159 | chitinase acidic | CHIA |
| 8179221 | 4.464653 | 10.61759 | 8.981042 | 4.94E-07 | 2.25E-04 | 6.690731 | 135656 | mucin like 3 | MUCL3 |
| 8098246 | 4.319376 | 9.852317 | 7.366769 | 4.61E-06 | 8.25E-04 | 4.53854 | 11199 | annexin A10 | ANXA10 |
| 8022655 | 4.254762 | 7.506952 | 10.92345 | 4.84E-08 | 5.33E-05 | 8.861758 | 361 | aquaporin 4 | AQP4 |
| 7924309 | 3.956132 | 7.763269 | 10.44772 | 8.28E-08 | 7.78E-05 | 8.367645 | 2104 | estrogen related receptor gamma | ESRRG |
| 8121936 | 3.850443 | 8.631479 | 4.629984 | 4.38E-04 | 0.011245 | 0.040841 | 352999 | chromosome 6 open reading frame 58 | C6orf58 |
| 8026877 | 3.841335 | 9.138758 | 5.899588 | 4.66E-05 | 0.003141 | 2.262675 | 6528 | solute carrier family 5 member 5 | SLC5A5 |
| 8147132 | 3.774964 | 11.6995 | 10.16004 | 1.16E-07 | 9.15E-05 | 8.05756 | 760 | carbonic anhydrase 2 | CA2 |
| 8046824 | 3.738866 | 7.985639 | 6.982016 | 8.22E-06 | 0.00115 | 3.972472 | 401024 | fibrous sheath interacting protein 2 | FSIP2 |
| 8121152 | 3.738046 | 8.007201 | 6.391405 | 2.08E-05 | 0.001934 | 3.061211 | 10690 | fucosyltransferase 9 | FUT9 |
| 8090955 | 3.664823 | 8.227204 | 7.239108 | 5.57E-06 | 8.97E-04 | 4.353094 | 51146 | alpha-1,4-N-acetylglucosaminyltransferase | A4GNT |
| 8138363 | 3.640977 | 8.926489 | 9.196439 | 3.75E-07 | 1.94E-04 | 6.952526 | 25928 | sclerostin domain containing 1 | SOSTDC1 |
| 8068383 | 3.600437 | 9.073651 | 8.963452 | 5.06E-07 | 2.25E-04 | 6.669103 | 54102 | chloride intracellular channel 6 | CLIC6 |
| 8086607 | 3.578003 | 8.573268 | 5.891122 | 4.73E-05 | 0.003168 | 2.248613 | 4057 | lactotransferrin | LTF |
| 8099746 | 3.567501 | 8.793245 | 6.099955 | 3.34E-05 | 0.002564 | 2.592361 | 886 | cholecystokinin A receptor | CCKAR |
| 8068583 | 3.507892 | 9.024796 | 7.026378 | 7.68E-06 | 0.001101 | 4.038837 | 3772 | potassium inwardly rectifying channel subfamily J member 15 | KCNJ15 |
| 8061894 | 3.499342 | 9.053731 | 5.076099 | 1.94E-04 | 0.006826 | 0.846337 | 92747 | BPI fold containing family B member 1 | BPIFB1 |
| 7909127 | 3.49705 | 9.728245 | 9.572271 | 2.35E-07 | 1.45E-04 | 7.396093 | 148808 | major facilitator superfamily domain containing 4A | MFSD4A |
| 8091799 | 3.471693 | 8.384558 | 5.77981 | 5.70E-05 | 0.003545 | 2.062744 | 165679 | serine palmitoyltransferase small subunit B | SPTSSB |
| 8103311 | 3.39834 | 6.914721 | 6.796864 | 1.09E-05 | 0.001317 | 3.692364 | 2243 | fibrinogen alpha chain | FGA |
| 8044212 | 3.388844 | 10.78908 | 12.8663 | 6.47E-09 | 1.78E-05 | 10.66187 | 6819 | sulfotransferase family 1C member 2 | SULT1C2 |
| 7909946 | 3.341746 | 8.344184 | 7.298958 | 5.09E-06 | 8.58E-04 | 4.440326 | 400823 | family with sequence similarity 177 member B | FAM177B |
| 8045664 | 3.310161 | 9.420512 | 7.989761 | 1.88E-06 | 5.02E-04 | 5.410525 | 130576 | LY6/PLAUR domain containing 6B | LYPD6B |
| 8148220 | 3.309982 | 9.475485 | 9.251336 | 3.50E-07 | 1.93E-04 | 7.018355 | 654463 | fer-1 like family member 6 | FER1L6 |
| 8112668 | 3.294048 | 8.467595 | 13.75896 | 2.80E-09 | 1.08E-05 | 11.38578 | 51301 | glucosaminyl (N-acetyl) transferase 4 | GCNT4 |
| 7954589 | 3.250626 | 7.478453 | 7.028723 | 7.65E-06 | 0.001101 | 4.042337 | 387849 | RAB15 effector protein | REP15 |
| 8009493 | 3.238542 | 7.309961 | 7.709539 | 2.79E-06 | 6.21E-04 | 5.024999 | 3773 | potassium inwardly rectifying channel subfamily J member 16 | KCNJ16 |
| 7990555 | 3.229763 | 7.911842 | 7.278018 | 5.26E-06 | 8.76E-04 | 4.409864 | 145957 | neuregulin 4 | NRG4 |
| 8136336 | 3.204449 | 10.5839 | 8.1111 | 1.58E-06 | 4.48E-04 | 5.574138 | 57016 | aldo-keto reductase family 1 member B10 | AKR1B10 |
| 8094988 | 3.20149 | 7.253217 | 5.564246 | 8.24E-05 | 0.00448 | 1.697623 | 80157 | cell wall biogenesis 43 C-terminal homolog | CWH43 |
| 8002283 | 3.172556 | 7.278777 | 5.436747 | 1.03E-04 | 0.005 | 1.4785 | 146456 | transmembrane p24 trafficking protein 6 | TMED6 |
| 8093950 | 3.073896 | 10.88457 | 5.331244 | 1.24E-04 | 0.005392 | 1.295431 | 6286 | S100 calcium binding protein P | S100P |
| 8056837 | 3.073076 | 8.819147 | 6.24023 | 2.65E-05 | 0.002238 | 2.819605 | 151556 | G protein-coupled receptor 155 | GPR155 |
| 8100827 | 3.070344 | 12.64207 | 5.503891 | 9.15E-05 | 0.004655 | 1.594186 | 3512 | joining chain of multimeric IgA and IgM | JCHAIN |
| 8029136 | 3.012382 | 9.811041 | 7.859132 | 2.26E-06 | 5.57E-04 | 5.232151 | 973 | CD79a molecule | CD79A |
| 7981996 | 2.986653 | 10.50495 | 8.958206 | 5.09E-07 | 2.25E-04 | 6.662646 | 1E+08 | small nucleolar RNA, C/D box 116-24 | SNORD116-24 |
| 8099255 | 2.961903 | 8.582306 | 5.279322 | 1.35E-04 | 0.005621 | 1.204763 | 768239 | prosaposin like 1 | PSAPL1 |
| 8043504 | 2.960749 | 9.23093 | 4.866302 | 2.84E-04 | 0.008716 | 0.470593 | 4118 | mal, T cell differentiation protein | MAL |
| 8081826 | 2.93038 | 8.04807 | 5.519478 | 8.90E-05 | 0.00463 | 1.620948 | 7348 | uroplakin 1B | UPK1B |
| 7910466 | 2.913727 | 8.884672 | 6.812635 | 1.07E-05 | 0.001295 | 3.716421 | 10753 | calpain 9 | CAPN9 |
| 7954208 | 2.9028 | 8.681175 | 5.072804 | 1.96E-04 | 0.006843 | 0.840481 | 5288 | phosphatidylinositol-4-phosphate 3-kinase catalytic subunit type 2 gamma | PIK3C2G |
| 7999909 | 2.82437 | 10.37709 | 7.567386 | 3.43E-06 | 6.85E-04 | 4.825267 | 51704 | G protein-coupled receptor class C group 5 member B | GPRC5B |
| 8111677 | 2.813674 | 8.600391 | 5.843367 | 5.12E-05 | 0.003314 | 2.169096 | 3977 | LIF receptor subunit alpha | LIFR |
| 7901883 | 2.736488 | 5.294804 | 5.686438 | 6.68E-05 | 0.003963 | 1.905424 | 27329 | angiopoietin like 3 | ANGPTL3 |
| 7975284 | 2.707629 | 8.215963 | 4.855256 | 2.90E-04 | 0.008808 | 0.450654 | 145226 | retinol dehydrogenase 12 | RDH12 |
| 8130578 | 2.705505 | 10.31123 | 5.368871 | 1.16E-04 | 0.005225 | 1.360902 | 677806 | small nucleolar RNA, H/ACA box 20 | SNORA20 |
| 8162492 | 2.647634 | 7.2081 | 8.622133 | 7.91E-07 | 2.87E-04 | 6.24181 | 8789 | fructose-bisphosphatase 2 | FBP2 |
| 7904361 | 2.62251 | 9.463261 | 10.27247 | 1.01E-07 | 8.94E-05 | 8.179783 | 54855 | terminal nucleotidyltransferase 5C | TENT5C |
| 8131069 | 2.591961 | 8.557503 | 6.903295 | 9.27E-06 | 0.001204 | 3.853995 | 2852 | G protein-coupled estrogen receptor 1 | GPER1 |
| 8155083 | 2.590224 | 10.36395 | 5.951729 | 4.27E-05 | 0.003006 | 2.349042 | 768 | carbonic anhydrase 9 | CA9 |
| 8127072 | 2.584532 | 9.065487 | 4.346031 | 7.43E-04 | 0.015468 | -0.48371 | 2938 | glutathione S-transferase alpha 1 | GSTA1 |
| 7988350 | 2.521415 | 10.08261 | 3.645486 | 0.002836 | 0.033487 | -1.8051 | 50506 | dual oxidase 2 | DUOX2 |
| 7981949 | 2.507339 | 8.016904 | 6.288869 | 2.45E-05 | 0.002147 | 2.897712 | 1E+08 | small nucleolar RNA, C/D box 116-1 | SNORD116-1 |
| 8046833 | 2.492568 | 6.197316 | 5.903054 | 4.63E-05 | 0.003132 | 2.268427 | 401024 | fibrous sheath interacting protein 2 | FSIP2 |
| 8106689 | 2.45856 | 7.88753 | 9.121279 | 4.13E-07 | 2.05E-04 | 6.861814 | 1160 | creatine kinase, mitochondrial 2 | CKMT2 |
| 8135774 | 2.442755 | 6.586043 | 5.088815 | 1.90E-04 | 0.006748 | 0.868927 | 5803 | protein tyrosine phosphatase receptor type Z1 | PTPRZ1 |
| 8114964 | 2.435997 | 10.45523 | 4.986802 | 2.28E-04 | 0.007598 | 0.6871 | 6690 | serine peptidase inhibitor Kazal type 1 | SPINK1 |
| 8145293 | 2.423179 | 10.05001 | 8.934557 | 5.25E-07 | 2.25E-04 | 6.633492 | 10863 | ADAM metallopeptidase domain 28 | ADAM28 |
| 7938090 | 2.410826 | 8.635009 | 6.899103 | 9.33E-06 | 0.001204 | 3.847661 | 887 | cholecystokinin B receptor | CCKBR |
| 7981994 | 2.382868 | 7.278888 | 7.165785 | 6.22E-06 | 9.68E-04 | 4.245518 | 1E+08 | small nucleolar RNA, C/D box 116-23 | SNORD116-23 |
| 8092682 | 2.379044 | 9.931717 | 5.127314 | 1.77E-04 | 0.006546 | 0.937192 | 6750 | somatostatin | SST |
| 7907260 | 2.377461 | 6.8453 | 3.757462 | 0.002283 | 0.029335 | -1.59225 | 388714 | flavin containing dimethylaniline monoxygenase 6, pseudogene | FMO6P |
| 8108627 | 2.374179 | 5.71957 | 3.848997 | 0.001914 | 0.026747 | -1.41854 | 56664 | vault RNA 1-1 | VTRNA1-1 |
| 8065948 | 2.343612 | 9.010395 | 4.702691 | 3.83E-04 | 0.010399 | 0.173759 | 80307 | fer-1 like family member 4 (pseudogene) | FER1L4 |
| 8040073 | 2.30719 | 7.005532 | 7.901664 | 2.13E-06 | 5.37E-04 | 5.290483 | 150622 | sciatic injury induced lincRNA upregulator of SOX11 | SILC1 |
| 7999291 | 2.298198 | 9.116978 | 4.537318 | 5.20E-04 | 0.012517 | -0.12942 | 146556 | chromosome 16 open reading frame 89 | C16orf89 |
| 8103769 | 2.294609 | 10.7994 | 5.52733 | 8.78E-05 | 0.004606 | 1.634418 | 3248 | 15-hydroxyprostaglandin dehydrogenase | HPGD |
| 8146687 | 2.285948 | 7.574096 | 5.822918 | 5.30E-05 | 0.003391 | 2.134943 | 137872 | alcohol dehydrogenase iron containing 1 | ADHFE1 |
| 7983405 | 2.262315 | 8.814974 | 4.297686 | 8.14E-04 | 0.016279 | -0.57381 | 405753 | dual oxidase maturation factor 2 | DUOXA2 |
| 8171725 | 2.26061 | 6.772569 | 6.545507 | 1.62E-05 | 0.001657 | 3.303978 | 256714 | MAP7 domain containing 2 | MAP7D2 |
| 7956658 | 2.260249 | 8.171602 | 6.446937 | 1.90E-05 | 0.001829 | 3.149103 | 9194 | solute carrier family 16 member 7 | SLC16A7 |
| 8043443 | 2.260019 | 11.03408 | 4.678619 | 4.00E-04 | 0.010622 | 0.129819 | 28923 | immunoglobulin kappa variable 2-24 | IGKV2-24 |
| 7981976 | 2.244507 | 10.80933 | 6.621484 | 1.44E-05 | 0.001538 | 3.422365 | 1E+08 | small nucleolar RNA, C/D box 116-14 | SNORD116-14 |
| 8083246 | 2.241651 | 6.218694 | 4.109198 | 0.001163 | 0.019942 | -0.92698 | 1360 | carboxypeptidase B1 | CPB1 |
| 7918794 | 2.228159 | 7.074798 | 8.399371 | 1.07E-06 | 3.69E-04 | 5.954964 | 270 | adenosine monophosphate deaminase 1 | AMPD1 |
| 7993267 | 2.214499 | 6.369652 | 6.914404 | 9.12E-06 | 0.001204 | 3.87077 | 608 | TNF receptor superfamily member 17 | TNFRSF17 |
| 7918457 | 2.213499 | 7.76469 | 6.700993 | 1.27E-05 | 0.001415 | 3.545335 | 3738 | potassium voltage-gated channel subfamily A member 3 | KCNA3 |
| 7925342 | 2.201825 | 7.48848 | 4.876631 | 2.79E-04 | 0.008665 | 0.489223 | 56605 | endoplasmic reticulum oxidoreductase 1 beta | ERO1B |
| 7920642 | 2.180154 | 11.77227 | 9.93758 | 1.51E-07 | 1.11E-04 | 7.8117 | 4582 | mucin 1, cell surface associated | MUC1 |
| 8171624 | 2.15566 | 7.228373 | 5.285687 | 1.34E-04 | 0.005566 | 1.215899 | 10149 | adhesion G protein-coupled receptor G2 | ADGRG2 |
| 7976322 | 2.139094 | 8.207992 | 5.898879 | 4.66E-05 | 0.003141 | 2.261497 | 1113 | chromogranin A | CHGA |
| 8110685 | 2.136365 | 9.239935 | 4.754796 | 3.48E-04 | 0.009813 | 0.26864 | 25845 | uncharacterized LOC25845 | PP7080 |
| 8084165 | 2.13086 | 7.637459 | 6.460997 | 1.86E-05 | 0.001819 | 3.171283 | 6657 | SRY-box transcription factor 2 | SOX2 |
| 8092800 | 2.128875 | 7.101591 | 6.722162 | 1.23E-05 | 0.001393 | 3.577918 | 84239 | ATPase 13A4 | ATP13A4 |
| 8102249 | 2.125923 | 6.05972 | 5.298778 | 1.31E-04 | 0.005552 | 1.238782 | 64850 | ethanolamine-phosphate phospho-lyase | ETNPPL |
| 8110971 | 2.109664 | 9.582282 | 4.424878 | 6.41E-04 | 0.014004 | -0.33724 | 134147 | carboxymethylenebutenolidase homolog | CMBL |
| 7981960 | 2.107877 | 7.25994 | 5.651446 | 7.10E-05 | 0.004045 | 1.846139 | 1E+08 | small nucleolar RNA, C/D box 116-6 | SNORD116-6 |
| 7991034 | 2.10166 | 8.048531 | 5.698963 | 6.54E-05 | 0.003902 | 1.926602 | 9455 | homer scaffold protein 2 | HOMER2 |
| 8161755 | 2.100248 | 10.73826 | 7.324637 | 4.90E-06 | 8.40E-04 | 4.477597 | 216 | aldehyde dehydrogenase 1 family member A1 | ALDH1A1 |
| 8101904 | 2.076779 | 6.645513 | 4.38445 | 6.91E-04 | 0.01473 | -0.41227 | 131 | alcohol dehydrogenase 7 (class IV), mu or sigma polypeptide | ADH7 |
| 8000799 | 2.065631 | 9.326659 | 4.780086 | 3.32E-04 | 0.009553 | 0.314574 | 79153 | glycerophosphodiester phosphodiesterase domain containing 3 | GDPD3 |
| 7981978 | 2.058332 | 12.05414 | 7.744962 | 2.66E-06 | 6.09E-04 | 5.074332 | 1E+08 | small nucleolar RNA, C/D box 116-15 | SNORD116-15 |
| 7967028 | 2.052121 | 9.134596 | 3.696131 | 0.002571 | 0.031643 | -1.7088 | 26834 | RNA, U4 small nuclear 2 | RNU4-2 |
| 8028084 | 2.049569 | 8.144093 | 7.02239 | 7.73E-06 | 0.001103 | 4.032883 | 333 | amyloid beta precursor like protein 1 | APLP1 |
| 8110688 | 2.048505 | 8.640794 | 5.310729 | 1.28E-04 | 0.005504 | 1.259652 | 6550 | solute carrier family 9 member A3 | SLC9A3 |
| 7999952 | 2.04642 | 6.623838 | 5.111263 | 1.83E-04 | 0.006622 | 0.908756 | 204474 | protein disulfide isomerase like, testis expressed | PDILT |
| 8085293 | 2.030829 | 8.73623 | 6.042856 | 3.67E-05 | 0.002697 | 2.499017 | 51738 | ghrelin and obestatin prepropeptide | GHRL |
| 7986639 | 2.026001 | 10.77682 | 4.547189 | 5.10E-04 | 0.012346 | -0.11124 | 1.03E+08 | putative V-set and immunoglobulin domain-containing-like protein IGHV4OR15-8 | LOC102724971 |
| 8062490 | 2.017008 | 9.15164 | 3.894343 | 0.001754 | 0.025656 | -1.33261 | 677837 | small nucleolar RNA, H/ACA box 60 | SNORA60 |
| 8083415 | 2.014954 | 7.787005 | 4.071544 | 0.001249 | 0.020778 | -0.99786 | 13 | arylacetamide deacetylase | AADAC |
| 7981427 | 2.008265 | 10.11915 | 5.005046 | 2.21E-04 | 0.007409 | 0.719718 | 1152 | creatine kinase B | CKB |
| 7933640 | -2.00057 | 7.509779 | -4.87252 | 2.81E-04 | 0.008681 | 0.481805 | 29974 | APOBEC1 complementation factor | A1CF |
| 7913216 | -2.02657 | 7.884391 | -3.49366 | 0.003808 | 0.039801 | -2.09397 | 5320 | phospholipase A2 group IIA | PLA2G2A |
| 8095736 | -2.03054 | 8.050784 | -3.97992 | 0.001488 | 0.023063 | -1.17072 | 374 | amphiregulin | AREG |
| 8138466 | -2.03441 | 8.550689 | -4.58736 | 4.74E-04 | 0.011871 | -0.03736 | 346389 | MET transcriptional regulator MACC1 | MACC1 |
| 8098439 | -2.05683 | 11.18974 | -9.77247 | 1.84E-07 | 1.23E-04 | 7.62571 | 4072 | epithelial cell adhesion molecule | EPCAM |
| 7957140 | -2.06887 | 7.496119 | -4.61672 | 4.49E-04 | 0.011439 | 0.016518 | 8549 | leucine rich repeat containing G protein-coupled receptor 5 | LGR5 |
| 8123104 | -2.11499 | 6.602816 | -3.69038 | 0.0026 | 0.031883 | -1.71973 | 84624 | fibronectin type III domain containing 1 | FNDC1 |
| 8091723 | -2.12719 | 7.158453 | -9.27506 | 3.40E-07 | 1.93E-04 | 7.046691 | 5918 | retinoic acid receptor responder 1 | RARRES1 |
| 8059376 | -2.129 | 7.765145 | -7.36674 | 4.61E-06 | 8.25E-04 | 4.538492 | 5270 | serpin family E member 2 | SERPINE2 |
| 7913566 | -2.14258 | 7.656311 | -7.47142 | 3.95E-06 | 7.61E-04 | 4.688818 | 3352 | 5-hydroxytryptamine receptor 1D | HTR1D |
| 8059905 | -2.15622 | 8.398731 | -4.40577 | 6.64E-04 | 0.014345 | -0.37268 | 1293 | collagen type VI alpha 3 chain | COL6A3 |
| 8129562 | -2.16511 | 9.134062 | -4.3688 | 7.12E-04 | 0.015003 | -0.44135 | 1490 | cellular communication network factor 2 | CCN2 |
| 8002891 | -2.17022 | 8.139629 | -4.37489 | 7.04E-04 | 0.014893 | -0.43002 | 23563 | carbohydrate sulfotransferase 5 | CHST5 |
| 7951284 | -2.17479 | 6.740023 | -4.49983 | 5.57E-04 | 0.012994 | -0.19857 | 4314 | matrix metallopeptidase 3 | MMP3 |
| 7924987 | -2.18698 | 8.176115 | -9.59059 | 2.29E-07 | 1.44E-04 | 7.417289 | 183 | angiotensinogen | AGT |
| 7996819 | -2.21775 | 7.212819 | -6.72476 | 1.22E-05 | 0.001392 | 3.581905 | 1001 | cadherin 3 | CDH3 |
| 8137271 | -2.23569 | 8.521472 | -7.8472 | 2.30E-06 | 5.57E-04 | 5.215744 | 26 | amine oxidase copper containing 1 | AOC1 |
| 7898809 | -2.24181 | 7.708145 | -6.84379 | 1.02E-05 | 0.00125 | 3.76383 | 2048 | EPH receptor B2 | EPHB2 |
| 8115623 | -2.2536 | 8.94108 | -5.42512 | 1.05E-04 | 0.005047 | 1.458409 | 23120 | ATPase phospholipid transporting 10B (putative) | ATP10B |
| 8056257 | -2.26262 | 5.561426 | -4.53768 | 5.19E-04 | 0.012517 | -0.12875 | 2191 | fibroblast activation protein alpha | FAP |
| 8160504 | -2.27427 | 4.454414 | -4.75865 | 3.45E-04 | 0.009777 | 0.275649 | 64922 | leucine rich repeat containing 19 | LRRC19 |
| 8096704 | -2.27964 | 9.272325 | -11.2675 | 3.32E-08 | 4.45E-05 | 9.205235 | 255743 | nephronectin | NPNT |
| 8020762 | -2.28328 | 6.57774 | -4.11363 | 0.001153 | 0.019819 | -0.91864 | 1830 | desmoglein 3 | DSG3 |
| 7954090 | -2.30541 | 9.109184 | -6.69201 | 1.29E-05 | 0.001415 | 3.531491 | 2012 | epithelial membrane protein 1 | EMP1 |
| 8096301 | -2.32692 | 6.932031 | -8.2249 | 1.35E-06 | 3.98E-04 | 5.725791 | 6696 | secreted phosphoprotein 1 | SPP1 |
| 8144866 | -2.36074 | 5.546536 | -4.54877 | 5.09E-04 | 0.012342 | -0.10834 | 10 | N-acetyltransferase 2 | NAT2 |
| 8038683 | -2.3829 | 6.843924 | -3.94105 | 0.001604 | 0.024249 | -1.2442 | 5653 | kallikrein related peptidase 6 | KLK6 |
| 8038725 | -2.38515 | 7.955342 | -5.41141 | 1.07E-04 | 0.005071 | 1.434681 | 5655 | kallikrein related peptidase 10 | KLK10 |
| 8058765 | -2.41086 | 9.354292 | -4.09922 | 0.001185 | 0.020145 | -0.94574 | 2335 | fibronectin 1 | FN1 |
| 8170648 | -2.42052 | 9.735088 | -5.59588 | 7.81E-05 | 0.004321 | 1.751626 | 633 | biglycan | BGN |
| 8056222 | -2.42461 | 7.895786 | -3.55476 | 0.003382 | 0.036869 | -1.97772 | 1803 | dipeptidyl peptidase 4 | DPP4 |
| 8095744 | -2.42738 | 7.755288 | -3.78243 | 0.002176 | 0.028488 | -1.54483 | 374 | amphiregulin | AREG |
| 8142171 | -2.4696 | 6.848386 | -4.16062 | 0.001055 | 0.018776 | -0.83036 | 1811 | solute carrier family 26 member 3 | SLC26A3 |
| 8139207 | -2.47006 | 7.290131 | -6.27535 | 2.51E-05 | 0.002182 | 2.876044 | 3624 | inhibin subunit beta A | INHBA |
| 7983650 | -2.49586 | 7.373537 | -5.80594 | 5.46E-05 | 0.00344 | 2.106545 | 11001 | solute carrier family 27 member 2 | SLC27A2 |
| 8013536 | -2.51188 | 7.384032 | -5.78727 | 5.63E-05 | 0.003514 | 2.075255 | 4843 | nitric oxide synthase 2 | NOS2 |
| 7954330 | -2.53627 | 4.397724 | -4.45082 | 6.10E-04 | 0.013611 | -0.28918 | 28234 | solute carrier organic anion transporter family member 1B3 | SLCO1B3 |
| 7913237 | -2.55056 | 10.43323 | -6.69462 | 1.28E-05 | 0.001415 | 3.535513 | 55450 | calcium/calmodulin dependent protein kinase II inhibitor 1 | CAMK2N1 |
| 8112731 | -2.57384 | 8.179484 | -5.37587 | 1.14E-04 | 0.005215 | 1.373054 | 2151 | coagulation factor II thrombin receptor like 2 | F2RL2 |
| 8139640 | -2.57927 | 7.712598 | -9.22886 | 3.60E-07 | 1.93E-04 | 6.99145 | 1644 | dopa decarboxylase | DDC |
| 8139087 | -2.60025 | 5.659013 | -3.28748 | 0.00569 | 0.04984 | -2.48579 | 6424 | secreted frizzled related protein 4 | SFRP4 |
| 8127563 | -2.66651 | 8.20776 | -6.00075 | 3.93E-05 | 0.002816 | 2.429871 | 1303 | collagen type XII alpha 1 chain | COL12A1 |
| 8161884 | -2.67503 | 6.729009 | -8.03429 | 1.76E-06 | 4.90E-04 | 5.470796 | 158471 | prune homolog 2 with BCH domain | PRUNE2 |
| 7954631 | -2.71491 | 8.226191 | -8.34073 | 1.16E-06 | 3.81E-04 | 5.878379 | 55711 | fatty acyl-CoA reductase 2 | FAR2 |
| 7982597 | -2.71709 | 9.248025 | -6.8914 | 9.45E-06 | 0.001209 | 3.836008 | 7057 | thrombospondin 1 | THBS1 |
| 8166769 | -2.72907 | 6.551398 | -6.08409 | 3.43E-05 | 0.002605 | 2.566472 | 5009 | ornithine transcarbamylase | OTC |
| 7957092 | -2.73115 | 7.254722 | -7.17828 | 6.10E-06 | 9.65E-04 | 4.263909 | 196446 | myelin regulatory factor like | MYRFL |
| 8045030 | -2.75348 | 7.76421 | -6.34263 | 2.25E-05 | 0.00201 | 2.983637 | 4648 | myosin VIIB | MYO7B |
| 7953200 | -2.77335 | 8.983405 | -8.37524 | 1.10E-06 | 3.74E-04 | 5.923512 | 894 | cyclin D2 | CCND2 |
| 8029098 | -2.79048 | 9.824525 | -4.63317 | 4.35E-04 | 0.011189 | 0.046679 | 4680 | CEA cell adhesion molecule 6 | CEACAM6 |
| 7937016 | -2.79251 | 6.63067 | -8.76291 | 6.57E-07 | 2.59E-04 | 6.41981 | 119467 | clarin 3 | CLRN3 |
| 8122202 | -2.79743 | 7.88684 | -7.75609 | 2.61E-06 | 6.06E-04 | 5.089795 | 4602 | MYB proto-oncogene, transcription factor | MYB |
| 8146863 | -2.82207 | 7.637183 | -5.01935 | 2.15E-04 | 0.007244 | 0.745259 | 23213 | sulfatase 1 | SULF1 |
| 8140140 | -2.83697 | 8.926122 | -7.43215 | 4.18E-06 | 7.81E-04 | 4.632617 | 1365 | claudin 3 | CLDN3 |
| 8130867 | -2.86417 | 7.843742 | -4.91461 | 2.60E-04 | 0.008273 | 0.557611 | 7058 | thrombospondin 2 | THBS2 |
| 8133360 | -2.8942 | 7.946169 | -10.2309 | 1.06E-07 | 9.12E-05 | 8.134747 | 1364 | claudin 4 | CLDN4 |
| 8155898 | -2.8982 | 8.045358 | -7.38649 | 4.47E-06 | 8.16E-04 | 4.566982 | 5125 | proprotein convertase subtilisin/kexin type 5 | PCSK5 |
| 7930593 | -2.90623 | 7.721292 | -6.34491 | 2.24E-05 | 0.00201 | 2.987263 | 79949 | pleckstrin homology domain containing S1 | PLEKHS1 |
| 8110620 | -2.92316 | 6.834401 | -5.13585 | 1.75E-04 | 0.00651 | 0.952297 | 10917 | butyrophilin like 3 | BTNL3 |
| 8028963 | -3.00002 | 6.454101 | -8.00591 | 1.84E-06 | 5.01E-04 | 5.432409 | 1555 | cytochrome P450 family 2 subfamily B member 6 | CYP2B6 |
| 8021584 | -3.0409 | 7.509884 | -4.3416 | 7.49E-04 | 0.015555 | -0.49196 | 5268 | serpin family B member 5 | SERPINB5 |
| 7951271 | -3.11387 | 8.191299 | -5.26604 | 1.39E-04 | 0.005701 | 1.181507 | 4312 | matrix metallopeptidase 1 | MMP1 |
| 8048319 | -3.14729 | 8.526536 | -9.78832 | 1.80E-07 | 1.23E-04 | 7.643699 | 7429 | villin 1 | VIL1 |
| 8108251 | -3.20055 | 6.696747 | -3.33759 | 0.00516 | 0.047203 | -2.39067 | 4888 | neuropeptide Y receptor Y6 (pseudogene) | NPY6R |
| 7931108 | -3.21565 | 8.450887 | -6.62304 | 1.44E-05 | 0.001538 | 3.424787 | 1755 | deleted in malignant brain tumors 1 | DMBT1 |
| 8081298 | -3.27409 | 6.620221 | -5.65939 | 7.00E-05 | 0.004034 | 1.859618 | 84873 | adhesion G protein-coupled receptor G7 | ADGRG7 |
| 8135661 | -3.27441 | 7.421211 | -5.71943 | 6.32E-05 | 0.003803 | 1.961162 | 1080 | CF transmembrane conductance regulator | CFTR |
| 8151592 | -3.36527 | 6.788031 | -3.96075 | 0.001544 | 0.023663 | -1.20694 | 759 | carbonic anhydrase 1 | CA1 |
| 7961455 | -3.45614 | 6.583465 | -7.56991 | 3.42E-06 | 6.85E-04 | 4.828833 | 2984 | guanylate cyclase 2C | GUCY2C |
| 8072587 | -3.46178 | 8.513773 | -7.56441 | 3.45E-06 | 6.85E-04 | 4.821058 | 6523 | solute carrier family 5 member 1 | SLC5A1 |
| 7919067 | -3.47275 | 9.768799 | -4.99033 | 2.27E-04 | 0.007559 | 0.693404 | 83998 | regenerating family member 4 | REG4 |
| 8101862 | -3.53076 | 6.646523 | -5.7619 | 5.88E-05 | 0.003602 | 2.032666 | 130 | alcohol dehydrogenase 6 (class V) | ADH6 |
| 8102523 | -3.66867 | 6.186009 | -11.4051 | 2.86E-08 | 4.01E-05 | 9.339461 | 2169 | fatty acid binding protein 2 | FABP2 |
| 8048026 | -3.80663 | 7.46494 | -5.67559 | 6.81E-05 | 0.003983 | 1.887065 | 1373 | carbamoyl-phosphate synthase 1 | CPS1 |
| 7922029 | -3.82134 | 8.537739 | -8.50017 | 9.32E-07 | 3.26E-04 | 6.085555 | 10223 | glycoprotein A33 | GPA33 |
| 8136709 | -3.99947 | 7.265815 | -6.47787 | 1.81E-05 | 0.001786 | 3.19787 | 93432 | maltase-glucoamylase 2 (putative) | MGAM2 |
| 8120088 | -4.01206 | 7.467971 | -6.29412 | 2.43E-05 | 0.002137 | 2.90612 | 4224 | meprin A subunit alpha | MEP1A |
| 8167973 | -4.0197 | 7.322764 | -9.33582 | 3.15E-07 | 1.83E-04 | 7.118957 | 9843 | hephaestin | HEPH |
| 8092726 | -4.03984 | 8.318253 | -11.6642 | 2.17E-08 | 3.56E-05 | 9.58743 | 9076 | claudin 1 | CLDN1 |
| 8135031 | -4.27849 | 8.363986 | -4.42595 | 6.40E-04 | 0.013987 | -0.33525 | 10071 | mucin 12, cell surface associated | MUC12 |
| 8065412 | -4.44419 | 6.638361 | -10.4434 | 8.32E-08 | 7.78E-05 | 8.363066 | 1469 | cystatin SN | CST1 |
| 7991335 | -4.57241 | 9.790853 | -9.75054 | 1.89E-07 | 1.24E-04 | 7.60078 | 290 | alanyl aminopeptidase, membrane | ANPEP |
| 8012126 | -4.61704 | 9.349597 | -12.0167 | 1.51E-08 | 2.91E-05 | 9.915172 | 1366 | claudin 7 | CLDN7 |
| 8135033 | -4.72153 | 8.338894 | -4.9613 | 2.39E-04 | 0.007829 | 0.641439 | 10071 | mucin 12, cell surface associated | MUC12 |
| 8059525 | -4.79784 | 6.490775 | -7.55642 | 3.49E-06 | 6.85E-04 | 4.809736 | 79853 | transmembrane 4 L six family member 20 | TM4SF20 |
| 8091811 | -4.79885 | 7.005799 | -6.36665 | 2.16E-05 | 0.001989 | 3.021877 | 6476 | sucrase-isomaltase | SI |
| 8135048 | -5.43369 | 9.036398 | -10.4786 | 7.99E-08 | 7.78E-05 | 8.400465 | 140453 | mucin 17, cell surface associated | MUC17 |
| 7969288 | -5.75161 | 9.077474 | -7.63536 | 3.11E-06 | 6.61E-04 | 4.921129 | 10562 | olfactomedin 4 | OLFM4 |
| 8151795 | -6.11941 | 8.174092 | -11.6428 | 2.22E-08 | 3.56E-05 | 9.567131 | 1015 | cadherin 17 | CDH17 |

**Supplementary Table S1C.** List of DEGs extracted from GEO datasets of CAG and FG

| Probe ID | Log FC | Ave Expr | t-test | P- Value | Adj P-Val | B | ENTREZ ID | Gene Name | Gene Symbol |
| --- | --- | --- | --- | --- | --- | --- | --- | --- | --- |
| 7972923 | 5.265218158 | 10.14352 | 6.341049 | 2.25E-05 | 0.007159 | 3.008406 | 496 | ATPase H+/K+ transporting subunit beta | ATP4B |
| 7948433 | 5.025212296 | 10.04833 | 5.72483 | 6.26E-05 | 0.011855 | 2.044251 | 2694 | cobalamin binding intrinsic factor | CBLIF |
| 8036110 | 4.676019808 | 10.22334 | 6.881528 | 9.59E-06 | 0.004495 | 3.804526 | 495 | ATPase H+/K+ transporting subunit alpha | ATP4A |
| 8136187 | 4.497952 | 8.614565 | 7.047088 | 7.44E-06 | 0.003824 | 4.039236 | 1358 | carboxypeptidase A2 | CPA2 |
| 7903945 | 4.143078245 | 7.771694 | 4.424742 | 6.41E-04 | 0.034526 | -0.17779 | 27159 | chitinase acidic | CHIA |
| 8022655 | 4.13105352 | 7.506952 | 10.60585 | 6.91E-08 | 4.26E-04 | 8.159776 | 361 | aquaporin 4 | AQP4 |
| 7928982 | 3.325972356 | 10.17233 | 6.437148 | 1.93E-05 | 0.006537 | 3.153331 | 8513 | lipase F, gastric type | LIPF |
| 8068383 | 3.234398799 | 9.073651 | 8.052183 | 1.72E-06 | 0.00163 | 5.375219 | 54102 | chloride intracellular channel 6 | CLIC6 |
| 8099746 | 3.059654193 | 8.793245 | 5.231605 | 1.47E-04 | 0.017477 | 1.22975 | 886 | cholecystokinin A receptor | CCKAR |
| 8056837 | 3.037124852 | 8.819147 | 6.167228 | 2.99E-05 | 0.008302 | 2.74254 | 151556 | G protein-coupled receptor 155 | GPR155 |
| 7909127 | 2.879905899 | 9.728245 | 7.882998 | 2.18E-06 | 0.001819 | 5.160737 | 148808 | major facilitator superfamily domain containing 4A | MFSD4A |
| 8068369 | 2.841550194 | 8.83974 | 5.130264 | 1.76E-04 | 0.019031 | 1.057858 | 9992 | potassium voltage-gated channel subfamily E regulatory subunit 2 | KCNE2 |
| 7924309 | 2.770785933 | 7.763269 | 7.317345 | 4.96E-06 | 0.003014 | 4.413306 | 2104 | estrogen related receptor gamma | ESRRG |
| 8009493 | 2.747657303 | 7.309961 | 6.540959 | 1.63E-05 | 0.005677 | 3.308243 | 3773 | potassium inwardly rectifying channel subfamily J member 16 | KCNJ16 |
| 7901883 | 2.684266352 | 5.294804 | 5.57792 | 8.05E-05 | 0.013309 | 1.805555 | 27329 | angiopoietin like 3 | ANGPTL3 |
| 8103311 | 2.561587699 | 6.914721 | 5.123315 | 1.79E-04 | 0.019081 | 1.046017 | 2243 | fibrinogen alpha chain | FGA |
| 8068583 | 2.521881902 | 9.024796 | 5.05138 | 2.03E-04 | 0.020209 | 0.923027 | 3772 | potassium inwardly rectifying channel subfamily J member 15 | KCNJ15 |
| 7914342 | 2.365530348 | 7.072692 | 5.309337 | 1.28E-04 | 0.016646 | 1.360568 | 2170 | fatty acid binding protein 3 | FABP3 |
| 8129666 | 2.306667743 | 7.908913 | 4.663462 | 4.11E-04 | 0.02903 | 0.247467 | 154091 | solute carrier family 2 member 12 | SLC2A12 |
| 8102249 | 2.186682958 | 6.05972 | 5.450219 | 1.00E-04 | 0.014958 | 1.595354 | 64850 | ethanolamine-phosphate phospho-lyase | ETNPPL |
| 8110685 | 2.107224097 | 9.239935 | 4.689939 | 3.92E-04 | 0.028164 | 0.294207 | 25845 | uncharacterized LOC25845 | PP7080 |
| 8131069 | 2.073899957 | 8.557503 | 5.523518 | 8.84E-05 | 0.013724 | 1.716313 | 2852 | G protein-coupled estrogen receptor 1 | GPER1 |
| 8035083 | -2.007868239 | 6.924313 | -5.60892 | 7.63E-05 | 0.013121 | 1.856195 | 8529 | cytochrome P450 family 4 subfamily F member 2 | CYP4F2 |
| 8006655 | -2.010512616 | 9.193142 | -5.77951 | 5.70E-05 | 0.011497 | 2.132235 | 79154 | dehydrogenase/reductase 11 | DHRS11 |
| 8005879 | -2.036835249 | 7.52707 | -6.73887 | 1.20E-05 | 0.005015 | 3.598859 | 9058 | solute carrier family 13-member 2 | SLC13A2 |
| 7916112 | -2.041045037 | 6.380018 | -11.3811 | 2.94E-08 | 3.02E-04 | 8.858555 | 5865 | RAB3B, member RAS oncogene family | RAB3B |
| 7913237 | -2.082527263 | 10.43323 | -5.46614 | 9.77E-05 | 0.01462 | 1.621704 | 55450 | calcium/calmodulin dependent protein kinase II inhibitor 1 | CAMK2N1 |
| 8144866 | -2.085261497 | 5.546536 | -4.01796 | 0.001384 | 0.048437 | -0.91625 | 10 | N-acetyltransferase 2 | NAT2 |
| 7945475 | -2.106799417 | 8.823959 | -8.79894 | 6.27E-07 | 0.001073 | 6.27436 | 53841 | cadherin related family member 5 | CDHR5 |
| 8139640 | -2.132785739 | 7.712598 | -7.6313 | 3.13E-06 | 0.002412 | 4.833993 | 1644 | dopa decarboxylase | DDC |
| 7960821 | -2.193790685 | 7.664537 | -8.48035 | 9.57E-07 | 0.001466 | 5.900047 | 339 | apolipoprotein B mRNA editing enzyme catalytic subunit 1 | APOBEC1 |
| 8083494 | -2.195013037 | 7.267154 | -4.87794 | 2.78E-04 | 0.023633 | 0.623491 | 4311 | membrane metalloendopeptidase | MME |
| 7940323 | -2.196282527 | 7.961599 | -6.53948 | 1.64E-05 | 0.005677 | 3.30605 | 83661 | membrane spanning 4-domains A8 | MS4A8 |
| 8094361 | -2.244147909 | 6.591824 | -4.67164 | 4.05E-04 | 0.028759 | 0.261912 | 57733 | glucosylceramidase beta 3 (gene/pseudogene) | GBA3 |
| 8137271 | -2.254500059 | 8.521472 | -7.91322 | 2.09E-06 | 0.001791 | 5.199347 | 26 | amine oxidase copper containing 1 | AOC1 |
| 7935116 | -2.288662248 | 7.382666 | -7.21743 | 5.75E-06 | 0.003286 | 4.276311 | 5950 | retinol binding protein 4 | RBP4 |
| 7942603 | -2.292676771 | 7.27713 | -5.16634 | 1.65E-04 | 0.018485 | 1.119227 | 80168 | monoacylglycerol O-acyltransferase 2 | MOGAT2 |
| 8016523 | -2.297154894 | 6.462642 | -9.15949 | 3.93E-07 | 8.09E-04 | 6.681965 | 2695 | gastric inhibitory polypeptide | GIP |
| 7961455 | -2.310830046 | 6.583465 | -5.06136 | 2.00E-04 | 0.020076 | 0.940132 | 2984 | guanylate cyclase 2C | GUCY2C |
| 8167973 | -2.321929907 | 7.322764 | -5.39271 | 1.11E-04 | 0.015488 | 1.499879 | 9843 | hephaestin | HEPH |
| 8024754 | -2.323586881 | 8.026689 | -9.80356 | 1.77E-07 | 5.82E-04 | 7.369912 | 84699 | cAMP responsive element binding protein 3 like 3 | CREB3L3 |
| 7983650 | -2.332595103 | 7.373537 | -5.42614 | 1.05E-04 | 0.015182 | 1.555439 | 11001 | solute carrier family 27 member 2 | SLC27A2 |
| 7924403 | -2.359389347 | 4.723784 | -5.03202 | 2.10E-04 | 0.020333 | 0.889802 | 406969 | microRNA 194-1 | MIR194-1 |
| 7972461 | -2.374963839 | 6.623627 | -7.41265 | 4.30E-06 | 0.00295 | 4.542572 | 6564 | solute carrier family 15 member 1 | SLC15A1 |
| 8035648 | -2.387438093 | 8.940146 | -6.30485 | 2.39E-05 | 0.00724 | 2.953438 | 53345 | transmembrane 6 superfamily member 2 | TM6SF2 |
| 8138337 | -2.411829763 | 5.581494 | -4.16563 | 0.001045 | 0.042437 | -0.64642 | 392636 | alkylglycerol monooxygenase | AGMO |
| 8094028 | -2.421944522 | 7.531929 | -4.73962 | 3.58E-04 | 0.026764 | 0.381665 | 84740 | AFAP1 antisense RNA 1 | AFAP1-AS1 |
| 8054479 | -2.587517616 | 9.817666 | -8.37616 | 1.10E-06 | 0.001477 | 5.77467 | 7851 | mal, T cell differentiation protein like | MALL |
| 7937016 | -2.599925167 | 6.63067 | -8.15858 | 1.48E-06 | 0.001578 | 5.508026 | 119467 | clarin 3 | CLRN3 |
| 8100760 | -2.672377843 | 5.687004 | -4.48867 | 5.69E-04 | 0.033306 | -0.06325 | 79799 | UDP glucuronosyltransferase family 2 member A3 | UGT2A3 |
| 8104268 | -2.755177977 | 7.876573 | -5.21853 | 1.51E-04 | 0.017667 | 1.207664 | 340024 | solute carrier family 6 member 19 | SLC6A19 |
| 7924401 | -2.804936869 | 4.231511 | -5.37959 | 1.14E-04 | 0.015705 | 1.478031 | 406997 | microRNA 215 | MIR215 |
| 8083301 | -2.849647867 | 7.748617 | -5.01322 | 2.18E-04 | 0.02065 | 0.857489 | 7104 | transmembrane 4 L six family member 4 | TM4SF4 |
| 8081298 | -2.916329295 | 6.620221 | -5.041 | 2.07E-04 | 0.020253 | 0.905212 | 84873 | adhesion G protein-coupled receptor G7 | ADGRG7 |
| 7999920 | -2.941171966 | 8.202067 | -8.20635 | 1.39E-06 | 0.001578 | 5.567124 | 2813 | glycoprotein 2 | GP2 |
| 8056222 | -3.014750707 | 7.895786 | -4.41998 | 6.47E-04 | 0.034605 | -0.18635 | 1803 | dipeptidyl peptidase 4 | DPP4 |
| 8162884 | -3.036578077 | 9.524402 | -4.23679 | 9.13E-04 | 0.040354 | -0.51705 | 229 | aldolase, fructose-bisphosphate B | ALDOB |
| 8101862 | -3.079768128 | 6.646523 | -5.02592 | 2.13E-04 | 0.020493 | 0.879329 | 130 | alcohol dehydrogenase 6 (class V) | ADH6 |
| 8171449 | -3.085260421 | 7.661203 | -8.21476 | 1.37E-06 | 0.001578 | 5.577503 | 59272 | angiotensin converting enzyme 2 | ACE2 |
| 7957092 | -3.088954924 | 7.254722 | -8.11869 | 1.57E-06 | 0.001612 | 5.458418 | 196446 | myelin regulatory factor like | MYRFL |
| 8110620 | -3.128694498 | 6.834401 | -5.49696 | 9.26E-05 | 0.014063 | 1.672573 | 10917 | butyrophilin like 3 | BTNL3 |
| 7913216 | -3.147046376 | 7.884391 | -5.42527 | 1.05E-04 | 0.015182 | 1.553988 | 5320 | phospholipase A2 group IIA | PLA2G2A |
| 8050619 | -3.214626971 | 6.545053 | -6.31241 | 2.36E-05 | 0.00724 | 2.964935 | 338 | apolipoprotein B | APOB |
| 8072587 | -3.226517403 | 8.513773 | -7.05034 | 7.41E-06 | 0.003824 | 4.043797 | 6523 | solute carrier family 5 member 1 | SLC5A1 |
| 8149142 | -3.278775446 | 6.796031 | -5.6497 | 7.12E-05 | 0.012684 | 1.922608 | 1670 | defensin alpha 5 | DEFA5 |
| 8101852 | -3.444803144 | 5.709294 | -4.44696 | 6.15E-04 | 0.033677 | -0.13794 | 127 | alcohol dehydrogenase 4 (class II), pi polypeptide | ADH4 |
| 8029098 | -3.538964812 | 9.824525 | -5.87591 | 4.85E-05 | 0.011197 | 2.286201 | 4680 | CEA cell adhesion molecule 6 | CEACAM6 |
| 8142171 | -3.540312996 | 6.848386 | -5.96447 | 4.18E-05 | 0.010111 | 2.426353 | 1811 | solute carrier family 26 member 3 | SLC26A3 |
| 8091071 | -3.579964904 | 7.220167 | -10.7977 | 5.57E-08 | 4.26E-04 | 8.338353 | 5948 | retinol binding protein 2 | RBP2 |
| 7922029 | -3.599558983 | 8.537739 | -8.00684 | 1.83E-06 | 0.001654 | 5.318143 | 10223 | glycoprotein A33 | GPA33 |
| 8048026 | -3.623917423 | 7.46494 | -5.40317 | 1.09E-04 | 0.015413 | 1.517277 | 1373 | carbamoyl-phosphate synthase 1 | CPS1 |
| 8048319 | -3.708253793 | 8.526536 | -11.533 | 2.50E-08 | 3.02E-04 | 8.988503 | 7429 | villin 1 | VIL1 |
| 8166769 | -3.777799487 | 6.551398 | -8.42209 | 1.03E-06 | 0.001466 | 5.830123 | 5009 | ornithine transcarbamylase | OTC |
| 8120088 | -3.833870386 | 7.467971 | -6.01458 | 3.85E-05 | 0.009522 | 2.505102 | 4224 | meprin A subunit alpha | MEP1A |
| 8012126 | -3.894869995 | 9.349597 | -10.1371 | 1.19E-07 | 5.82E-04 | 7.706998 | 1366 | claudin 7 | CLDN7 |
| 8021584 | -3.957358419 | 7.509884 | -5.65007 | 7.11E-05 | 0.012684 | 1.923207 | 5268 | serpin family B member 5 | SERPINB5 |
| 8135048 | -4.055998311 | 9.036398 | -7.82182 | 2.38E-06 | 0.001931 | 5.082165 | 140453 | mucin 17, cell surface associated | MUC17 |
| 8020827 | -4.14621776 | 6.121752 | -9.71352 | 1.97E-07 | 5.82E-04 | 7.276727 | 4225 | meprin A subunit beta | MEP1B |
| 8096580 | -4.262564455 | 6.533268 | -8.16096 | 1.48E-06 | 0.001578 | 5.510978 | 4547 | microsomal triglyceride transfer protein | MTTP |
| 8102523 | -4.369186941 | 6.186009 | -13.5829 | 3.29E-09 | 1.01E-04 | 10.54741 | 2169 | fatty acid binding protein 2 | FABP2 |
| 7991335 | -4.485065164 | 9.790853 | -9.56427 | 2.37E-07 | 5.82E-04 | 7.12016 | 290 | alanyl aminopeptidase, membrane | ANPEP |
| 7919067 | -4.580058786 | 9.768799 | -6.58153 | 1.53E-05 | 0.005669 | 3.368322 | 83998 | regenerating family member 4 | REG4 |
| 7931108 | -4.647932561 | 8.450887 | -9.573 | 2.34E-07 | 5.82E-04 | 7.12939 | 1755 | deleted in malignant brain tumors 1 | DMBT1 |
| 8059525 | -4.675468655 | 6.490775 | -7.36369 | 4.63E-06 | 0.003014 | 4.476335 | 79853 | transmembrane 4 L six family member 20 | TM4SF20 |
| 8151795 | -5.145005941 | 8.174092 | -9.78888 | 1.80E-07 | 5.82E-04 | 7.354777 | 1015 | cadherin 17 | CDH17 |
| 8069582 | -5.165785924 | 6.091056 | -6.57565 | 1.55E-05 | 0.005669 | 3.359636 | 5651 | transmembrane serine protease 15 | TMPRSS15 |
| 8091811 | -5.338291109 | 7.005799 | -7.08232 | 7.05E-06 | 0.003824 | 4.088641 | 6476 | sucrase-isomaltase | SI |
| 7902721 | -5.930032845 | 6.723051 | -5.82242 | 5.31E-05 | 0.011497 | 2.20094 | 1179 | chloride channel accessory 1 | CLCA1 |
| 7969288 | -6.018702394 | 9.077474 | -7.98993 | 1.88E-06 | 0.001654 | 5.29678 | 10562 | olfactomedin 4 | OLFM4 |

Supplementary Table S2. Common genes among three groups, intersection of FG-CAG-GC

| ID | Gene.symbol | Gene.title | log2(fold change)(GC vs FG) | -log10(Pvalue)(GC vs FG) | log2(fold change)(FG vs CAG) | -log10(Pvalue)(FG vs CAG) | log2(fold change)(CAG vs GC) | -log10(Pvalue)(CAG vs GC) |
| --- | --- | --- | --- | --- | --- | --- | --- | --- |
| 8092726 | CLDN1 | claudin 1 | 4.04 | 7.74 | -1.421 | 2.959 | -2.619 | 4.731 |
| 8133360 | CLDN4 | claudin 4 | 2.894 | 7.049 | -1.165 | 2.975 | -1.73 | 3.825 |
| 8096704 | NPNT | nephronectin | 2.28 | 7.575 | -1.134 | 4.175 | -1.145 | 3.533 |
| 8140129 | ABHD11 | abhydrolase domain containing 11 | 1.887 | 7.559 | -0.673 | 2.89 | -1.215 | 4.547 |
| 8141688 | PLOD3 | procollagen-lysine,2-oxoglutarate 5-dioxygenase 3 | 1.437 | 6.972 | -0.589 | 2.991 | -0.847 | 3.714 |
| 8141395 | MCM7 | minichromosome maintenance complex component 7 | 1.383 | 7.809 | -0.519 | 3.237 | -0.863 | 4.62 |
| 7922343 | TNFSF4 | tumor necrosis factor superfamily member 4 | 0.511 | 3.595 | 0.513 | 3.608 | -1.024 | 5.951 |
| 8019762 | P4HB | prolyl 4-hydroxylase subunit beta | -0.423 | 2.179 | -0.582 | 3.19 | 1.004 | 4.732 |
| 8034643 | CACNA1A | calcium voltage-gated channel subunit alpha1 A | -0.481 | 3.337 | -0.419 | 2.851 | 0.899 | 5.291 |
| 8085244 | CIDEC | cell death inducing DFFA like effector c | -0.619 | 2.611 | -1.622 | 6.749 | 2.241 | 7.565 |
| 8078971 | ENTPD3 | ectonucleoside triphosphate diphosphohydrolase 3 | -1.276 | 2.932 | -1.21 | 2.753 | 2.486 | 4.945 |
| 8074944 | DERL3 | derlin 3 | -1.779 | 7.049 | 0.825 | 3.49 | 0.954 | 3.404 |
| 8153334 |  |  | -3.113 | 6.815 | 1.329 | 3.024 | 1.784 | 3.485 |
| 8068369 | KCNE2 | potassium voltage-gated channel subfamily E regulatory subunit 2 | -5.587 | 6.963 | 2.842 | 3.787 | 2.746 | 3.028 |
| 7940421 | PGA4///PGA3///PGA5 | pepsinogen 4, group I (pepsinogen A)///pepsinogen 3, group I (pepsinogen A)///pepsinogen 5, group I (pepsinogen A) | -7.673 | 8.149 | 3.365 | 4.091 | 4.308 | 4.428 |
| 7940431 | PGA4///PGA3///PGA5 | pepsinogen 4, group I (pepsinogen A)///pepsinogen 3, group I (pepsinogen A)///pepsinogen 5, group I (pepsinogen A) | -7.759 | 8.233 | 3.405 | 4.158 | 4.354 | 4.493 |
| 7940441 | PGA4///PGA3///PGA5 | pepsinogen 4, group I (pepsinogen A)///pepsinogen 3, group I (pepsinogen A)///pepsinogen 5, group I (pepsinogen A) | -8.104 | 8.528 | 3.453 | 4.262 | 4.652 | 4.836 |
| 7928982 | LIPF | lipase F, gastric type | -9.489 | 10.245 | 3.326 | 4.758 | 6.163 | 6.96 |

**Supplementary Table S3.** Network topological features of giant network

| **Name** | **Avg Shortest Path length** | **Clustering coefficient** | **CC** | **Eccentricity** | **Stress** | **Degree** | **BC** | **Neighborhood conenctivity** | **Radiality** | **Topological coefficient** |
| --- | --- | --- | --- | --- | --- | --- | --- | --- | --- | --- |
| MCM7 | 1.522936 | 0.75735 | 0.656627 | 3 | 2964 | 70 | 0.00696 | 54.14286 | 0.992529 | 0.594976 |
| CDC6 | 1.522936 | 0.75735 | 0.656627 | 3 | 3090 | 70 | 0.007107 | 54.14286 | 0.992529 | 0.594976 |
| MCM2 | 1.53211 | 0.776641 | 0.652695 | 3 | 2956 | 69 | 0.006078 | 54.82609 | 0.992398 | 0.602484 |
| CDC45 | 1.53211 | 0.775789 | 0.652695 | 3 | 2962 | 69 | 0.006201 | 54.7971 | 0.992398 | 0.602166 |
| MCM4 | 1.541284 | 0.785777 | 0.64881 | 3 | 2872 | 68 | 0.00577 | 55.16176 | 0.992267 | 0.606173 |
| MCM3 | 1.550459 | 0.795568 | 0.64497 | 3 | 2648 | 67 | 0.005356 | 55.49254 | 0.992136 | 0.609808 |
| CDK1 | 1.522936 | 0.727273 | 0.656627 | 3 | 5822 | 67 | 0.038511 | 53.31343 | 0.992529 | 0.573102 |
| PCNA | 1.559633 | 0.776224 | 0.641176 | 3 | 2644 | 66 | 0.005979 | 54.86364 | 0.992005 | 0.602897 |
| RFC4 | 1.559633 | 0.807925 | 0.641176 | 3 | 2608 | 66 | 0.004834 | 55.87879 | 0.992005 | 0.614053 |
| CDK2 | 1.522936 | 0.715618 | 0.656627 | 3 | 6496 | 66 | 0.042891 | 52.86364 | 0.992529 | 0.556459 |
| MCM6 | 1.568807 | 0.817788 | 0.637427 | 3 | 2476 | 65 | 0.004675 | 56.24615 | 0.991874 | 0.61809 |
| MCM10 | 1.669725 | 0.804327 | 0.598901 | 4 | 1552 | 65 | 0.003556 | 55.61538 | 0.990433 | 0.670065 |
| RFC3 | 1.59633 | 0.805288 | 0.626437 | 4 | 2136 | 65 | 0.004193 | 55.84615 | 0.991481 | 0.627485 |
| MCM5 | 1.587156 | 0.803846 | 0.630058 | 4 | 2462 | 65 | 0.004898 | 55.72308 | 0.991612 | 0.619145 |
| CDT1 | 1.577982 | 0.779762 | 0.633721 | 3 | 2478 | 64 | 0.005692 | 55.03125 | 0.991743 | 0.604739 |
| POLD1 | 1.577982 | 0.829365 | 0.633721 | 3 | 2524 | 64 | 0.004431 | 56.60938 | 0.991743 | 0.622081 |
| CDC7 | 1.587156 | 0.811572 | 0.630058 | 3 | 2292 | 63 | 0.004701 | 56.11111 | 0.991612 | 0.616606 |
| POLA1 | 1.587156 | 0.824885 | 0.630058 | 3 | 2394 | 63 | 0.004473 | 56.49206 | 0.991612 | 0.620792 |
| ORC1 | 1.688073 | 0.813108 | 0.592391 | 4 | 1290 | 63 | 0.003236 | 55.85714 | 0.99017 | 0.672978 |
| RFC5 | 1.623853 | 0.828469 | 0.615819 | 4 | 2060 | 63 | 0.003604 | 56.53968 | 0.991088 | 0.642496 |
| POLE | 1.587156 | 0.838198 | 0.630058 | 3 | 2300 | 63 | 0.004003 | 56.96825 | 0.991612 | 0.626025 |
| RPA1 | 1.614679 | 0.803891 | 0.619318 | 4 | 2360 | 63 | 0.004495 | 55.73016 | 0.991219 | 0.626182 |
| WDHD1 | 1.697248 | 0.837123 | 0.589189 | 4 | 1168 | 62 | 0.002328 | 56.70968 | 0.990039 | 0.683249 |
| RPA2 | 1.623853 | 0.822845 | 0.615819 | 4 | 2148 | 62 | 0.003677 | 56.5 | 0.991088 | 0.634831 |
| TIPIN | 1.706422 | 0.816393 | 0.586022 | 4 | 1508 | 61 | 0.006862 | 56.03279 | 0.989908 | 0.675094 |
| CCNA2 | 1.59633 | 0.786441 | 0.626437 | 3 | 3406 | 60 | 0.015844 | 55.45 | 0.991481 | 0.596237 |
| POLA2 | 1.733945 | 0.862712 | 0.57672 | 4 | 966 | 60 | 0.001713 | 57.35 | 0.989515 | 0.69939 |
| RRM1 | 1.623853 | 0.79887 | 0.615819 | 4 | 4436 | 60 | 0.022941 | 55.9 | 0.991088 | 0.614286 |
| ATR | 1.642202 | 0.797175 | 0.608939 | 4 | 2042 | 60 | 0.007852 | 55.65 | 0.990826 | 0.625281 |
| PRIM1 | 1.733945 | 0.855932 | 0.57672 | 4 | 984 | 60 | 0.001773 | 57.1 | 0.989515 | 0.696341 |
| POLE2 | 1.715596 | 0.869492 | 0.582888 | 4 | 998 | 60 | 0.001741 | 57.66667 | 0.989777 | 0.694779 |
| RFC2 | 1.614679 | 0.815819 | 0.619318 | 3 | 2946 | 60 | 0.021788 | 56.08333 | 0.991219 | 0.622963 |
| TIMELESS | 1.752294 | 0.823956 | 0.570681 | 4 | 1256 | 58 | 0.005936 | 56.2931 | 0.989253 | 0.686501 |
| ATM | 1.623853 | 0.719903 | 0.615819 | 4 | 6156 | 58 | 0.049388 | 52.81034 | 0.991088 | 0.573838 |
| RPA3 | 1.669725 | 0.849624 | 0.598901 | 4 | 1582 | 57 | 0.002672 | 57.45614 | 0.990433 | 0.645575 |
| TOPBP1 | 1.642202 | 0.843985 | 0.608939 | 3 | 1790 | 57 | 0.003245 | 57.05263 | 0.990826 | 0.626952 |
| CHTF18 | 1.770642 | 0.855195 | 0.564767 | 4 | 858 | 56 | 0.001759 | 57.19643 | 0.988991 | 0.697517 |
| TP53 | 1.522936 | 0.534007 | 0.656627 | 3 | 40350 | 55 | 0.216334 | 46.4 | 0.992529 | 0.437736 |
| DTL | 1.688073 | 0.818855 | 0.592391 | 4 | 1480 | 55 | 0.003394 | 56.50909 | 0.99017 | 0.634934 |
| DNA2 | 1.688073 | 0.857239 | 0.592391 | 4 | 1364 | 55 | 0.002408 | 57.47273 | 0.99017 | 0.645761 |
| ORC6 | 1.779817 | 0.8633 | 0.561856 | 4 | 798 | 55 | 0.001678 | 57.32727 | 0.98886 | 0.699113 |
| DBF4 | 1.87156 | 0.865129 | 0.534314 | 4 | 612 | 54 | 0.001387 | 57.57407 | 0.987549 | 0.728786 |
| ORC2 | 1.889908 | 0.847659 | 0.529126 | 4 | 584 | 54 | 0.001586 | 57.07407 | 0.987287 | 0.731719 |
| PRIM2 | 1.807339 | 0.871419 | 0.553299 | 4 | 772 | 54 | 0.001362 | 57.38889 | 0.988467 | 0.717361 |
| ORC4 | 1.862385 | 0.846154 | 0.536946 | 4 | 684 | 53 | 0.00169 | 56.77358 | 0.98768 | 0.700908 |
| ORC5 | 1.899083 | 0.877828 | 0.52657 | 4 | 484 | 52 | 0.001246 | 57.92308 | 0.987156 | 0.733204 |
| GINS4 | 1.899083 | 0.904223 | 0.52657 | 4 | 454 | 52 | 8.38E-04 | 58.80769 | 0.987156 | 0.744401 |
| GINS1 | 1.807339 | 0.904977 | 0.553299 | 4 | 598 | 52 | 9.28E-04 | 58.42308 | 0.988467 | 0.712477 |
| ORC3 | 1.880734 | 0.872941 | 0.531707 | 4 | 524 | 51 | 0.001286 | 57.68627 | 0.987418 | 0.712176 |
| DSCC1 | 1.834862 | 0.879216 | 0.545 | 4 | 594 | 51 | 0.001103 | 57.98039 | 0.988073 | 0.715807 |
| RAD17 | 1.733945 | 0.861224 | 0.57672 | 4 | 942 | 50 | 0.002128 | 57.82 | 0.989515 | 0.649663 |
| GINS2 | 1.825688 | 0.911837 | 0.547739 | 4 | 508 | 50 | 8.01E-04 | 58.8 | 0.988204 | 0.717073 |
| CDC20 | 1.752294 | 0.85119 | 0.570681 | 4 | 994 | 49 | 0.00226 | 57.36735 | 0.989253 | 0.651902 |
| RRM2 | 1.669725 | 0.807823 | 0.598901 | 4 | 8064 | 49 | 0.045315 | 56.4898 | 0.990433 | 0.588223 |
| POLE3 | 1.926606 | 0.921099 | 0.519048 | 4 | 346 | 48 | 6.82E-04 | 59.25 | 0.986763 | 0.75 |
| OBFC1 | 1.752294 | 0.867908 | 0.570681 | 4 | 912 | 48 | 0.001672 | 58 | 0.989253 | 0.651685 |
| RFC1 | 1.917431 | 0.87234 | 0.521531 | 4 | 406 | 47 | 0.001005 | 57.40426 | 0.986894 | 0.735952 |
| ASF1B | 1.87156 | 0.913043 | 0.534314 | 4 | 286 | 46 | 5.66E-04 | 59.5 | 0.987549 | 0.734568 |
| GMNN | 1.761468 | 0.851515 | 0.567708 | 3 | 1256 | 45 | 0.002515 | 57.31111 | 0.989122 | 0.63679 |
| GINS3 | 1.899083 | 0.859408 | 0.52657 | 4 | 7364 | 44 | 0.042755 | 57.27273 | 0.987156 | 0.690033 |
| ATAD5 | 1.954128 | 0.900332 | 0.511737 | 4 | 270 | 43 | 5.56E-04 | 58.60465 | 0.98637 | 0.751342 |
| POLE4 | 1.990826 | 0.929152 | 0.502304 | 4 | 272 | 42 | 5.35E-04 | 59.80952 | 0.985845 | 0.766789 |
| RAD52 | 1.834862 | 0.905128 | 0.545 | 4 | 528 | 40 | 9.77E-04 | 58.325 | 0.988073 | 0.662784 |
| ATRIP | 1.844037 | 0.890688 | 0.542289 | 4 | 736 | 39 | 0.001116 | 58.74359 | 0.987942 | 0.667541 |
| MCMBP | 2.137615 | 0.968254 | 0.467811 | 4 | 58 | 36 | 8.64E-05 | 60.52778 | 0.983748 | 0.817943 |
| HUS1 | 1.798165 | 0.878788 | 0.556122 | 4 | 4320 | 34 | 0.021713 | 57.05882 | 0.988598 | 0.587932 |
| RRM2B | 1.889908 | 0.855615 | 0.529126 | 4 | 1012 | 34 | 0.004223 | 57.82353 | 0.987287 | 0.657086 |
| RAD9A | 1.899083 | 0.922348 | 0.52657 | 4 | 386 | 33 | 6.42E-04 | 58.09091 | 0.987156 | 0.660124 |
| SKP2 | 1.990826 | 0.905797 | 0.502304 | 4 | 110 | 24 | 3.27E-04 | 56.95833 | 0.985845 | 0.654693 |
| RBL1 | 2 | 0.853755 | 0.5 | 4 | 244 | 23 | 5.36E-04 | 56.56522 | 0.985714 | 0.650175 |
| CDKN1A | 2.027523 | 0.861905 | 0.493213 | 4 | 88 | 21 | 2.78E-04 | 54.61905 | 0.985321 | 0.627805 |
| CDKN1B | 2.045872 | 0.8 | 0.488789 | 4 | 420 | 21 | 7.33E-04 | 53.42857 | 0.985059 | 0.628571 |
| P4HB | 2.082569 | 0.45614 | 0.480176 | 4 | 9740 | 19 | 0.060761 | 14.52632 | 0.984535 | 0.181879 |
| PDIA3 | 2.229358 | 0.632353 | 0.44856 | 4 | 3548 | 17 | 0.01329 | 14.70588 | 0.982438 | 0.216263 |
| CALR | 2.229358 | 0.647059 | 0.44856 | 4 | 3478 | 17 | 0.01159 | 14.82353 | 0.982438 | 0.217993 |
| HSPA5 | 2.229358 | 0.647059 | 0.44856 | 4 | 3478 | 17 | 0.01159 | 14.82353 | 0.982438 | 0.217993 |
| CANX | 2.559633 | 0.6 | 0.390681 | 4 | 790 | 16 | 0.017531 | 12.3125 | 0.97772 | 0.371212 |
| HSP90B1 | 2.238532 | 0.675 | 0.446721 | 4 | 3520 | 16 | 0.013053 | 15.25 | 0.982307 | 0.224265 |
| EIF2AK3 | 1.963303 | 0.494505 | 0.509346 | 3 | 3880 | 14 | 0.037868 | 23.14286 | 0.986239 | 0.237113 |
| ERN1 | 2.266055 | 0.858974 | 0.441296 | 4 | 2042 | 13 | 0.005684 | 17.46154 | 0.981913 | 0.256787 |
| ATF6 | 2.266055 | 0.858974 | 0.441296 | 4 | 2042 | 13 | 0.005684 | 17.46154 | 0.981913 | 0.256787 |
| DNAJC10 | 2.807339 | 0.893939 | 0.356209 | 5 | 52 | 12 | 2.80E-04 | 14.08333 | 0.974181 | 0.612319 |
| ERO1L | 2.688073 | 0.963636 | 0.372014 | 4 | 78 | 11 | 2.97E-04 | 15 | 0.975885 | 0.576923 |
| DERL3 | 2.862385 | 0.963636 | 0.349359 | 5 | 18 | 11 | 1.20E-04 | 14.54545 | 0.973394 | 0.661157 |
| GJA1 | 2.082569 | 0.288889 | 0.480176 | 4 | 4144 | 10 | 0.032691 | 23.1 | 0.984535 | 0.251087 |
| PPIB | 2.110092 | 0.688889 | 0.473913 | 4 | 1736 | 10 | 0.008355 | 23.6 | 0.984142 | 0.268182 |
| MANF | 2.944954 | 1 | 0.339564 | 5 | 0 | 9 | 0 | 15 | 0.972215 | 0.714286 |
| TJP1 | 2.33945 | 0.535714 | 0.427451 | 4 | 6814 | 8 | 0.048961 | 12.5 | 0.980865 | 0.189394 |
| OCLN | 2.706422 | 0.5 | 0.369492 | 4 | 1296 | 8 | 0.014871 | 7.375 | 0.975623 | 0.268519 |
| KCNH2 | 2.321101 | 0.321429 | 0.43083 | 5 | 3430 | 8 | 0.032463 | 11.375 | 0.981127 | 0.163603 |
| CGN | 2.990826 | 0.571429 | 0.334356 | 5 | 296 | 7 | 0.002342 | 5.428571 | 0.97156 | 0.480519 |
| B2M | 2.348624 | 0.857143 | 0.425781 | 4 | 712 | 7 | 0.002407 | 19.71429 | 0.980734 | 0.303297 |
| TAPBP | 2.908257 | 0.933333 | 0.343849 | 5 | 4 | 6 | 5.66E-05 | 15.5 | 0.972739 | 0.704545 |
| APOB | 2.908257 | 0.8 | 0.343849 | 5 | 20 | 6 | 3.20E-04 | 14.83333 | 0.972739 | 0.674242 |
| KCNQ1 | 2.513761 | 0.6 | 0.39781 | 5 | 580 | 6 | 0.003537 | 12.5 | 0.978375 | 0.231481 |
| F11R | 3.229358 | 1 | 0.309659 | 5 | 0 | 5 | 0 | 6.6 | 0.968152 | 0.66 |
| CLDN4 | 3.229358 | 1 | 0.309659 | 5 | 0 | 5 | 0 | 6.6 | 0.968152 | 0.66 |
| CLDN1 | 3.229358 | 1 | 0.309659 | 5 | 0 | 5 | 0 | 6.6 | 0.968152 | 0.66 |
| YBX3 | 2.311927 | 0.6 | 0.43254 | 4 | 1376 | 5 | 0.011532 | 19.8 | 0.981258 | 0.253846 |
| AKAP9 | 2.348624 | 0.5 | 0.425781 | 4 | 954 | 5 | 0.005995 | 17.8 | 0.980734 | 0.244444 |
| CRY2 | 2.275229 | 0.5 | 0.439516 | 4 | 72 | 4 | 4.67E-04 | 48.25 | 0.981782 | 0.615385 |
| MTTP | 2.990826 | 0.833333 | 0.334356 | 5 | 2 | 4 | 2.83E-05 | 14.5 | 0.97156 | 0.690476 |
| KCNE1 | 2.944954 | 0.833333 | 0.339564 | 5 | 20 | 4 | 2.10E-04 | 7.25 | 0.972215 | 0.517857 |
| KCNE2 | 2.944954 | 0.833333 | 0.339564 | 5 | 20 | 4 | 2.10E-04 | 7.25 | 0.972215 | 0.517857 |
| ENTPD3 | 2.495413 | 1 | 0.400735 | 5 | 0 | 3 | 0 | 47.66667 | 0.978637 | 0.744792 |
| CACNA1A | 3.211009 | 0 | 0.311429 | 6 | 46 | 2 | 9.27E-04 | 7.5 | 0.968414 | 0.5 |
| TNFSF4 | 2.59633 | 0 | 0.385159 | 5 | 1776 | 2 | 0.018349 | 29.5 | 0.977195 | 0.5 |
| TNFRSF4 | 3.587156 | 0 | 0.278772 | 6 | 0 | 1 | 0 | 2 | 0.963041 | 0 |
| PLOD3 | 3.073394 | 0 | 0.325373 | 5 | 0 | 1 | 0 | 19 | 0.97038 | 0 |
| ABHD11 | 2.605505 | 0 | 0.383803 | 4 | 0 | 1 | 0 | 60 | 0.977064 | 0 |

**Supplementary Figure S1.** Functional enrichment analysis of GO terms for DEGs using ShinyGO tool. (A) Biological process (B) Cellular Components and (C) Molecular function


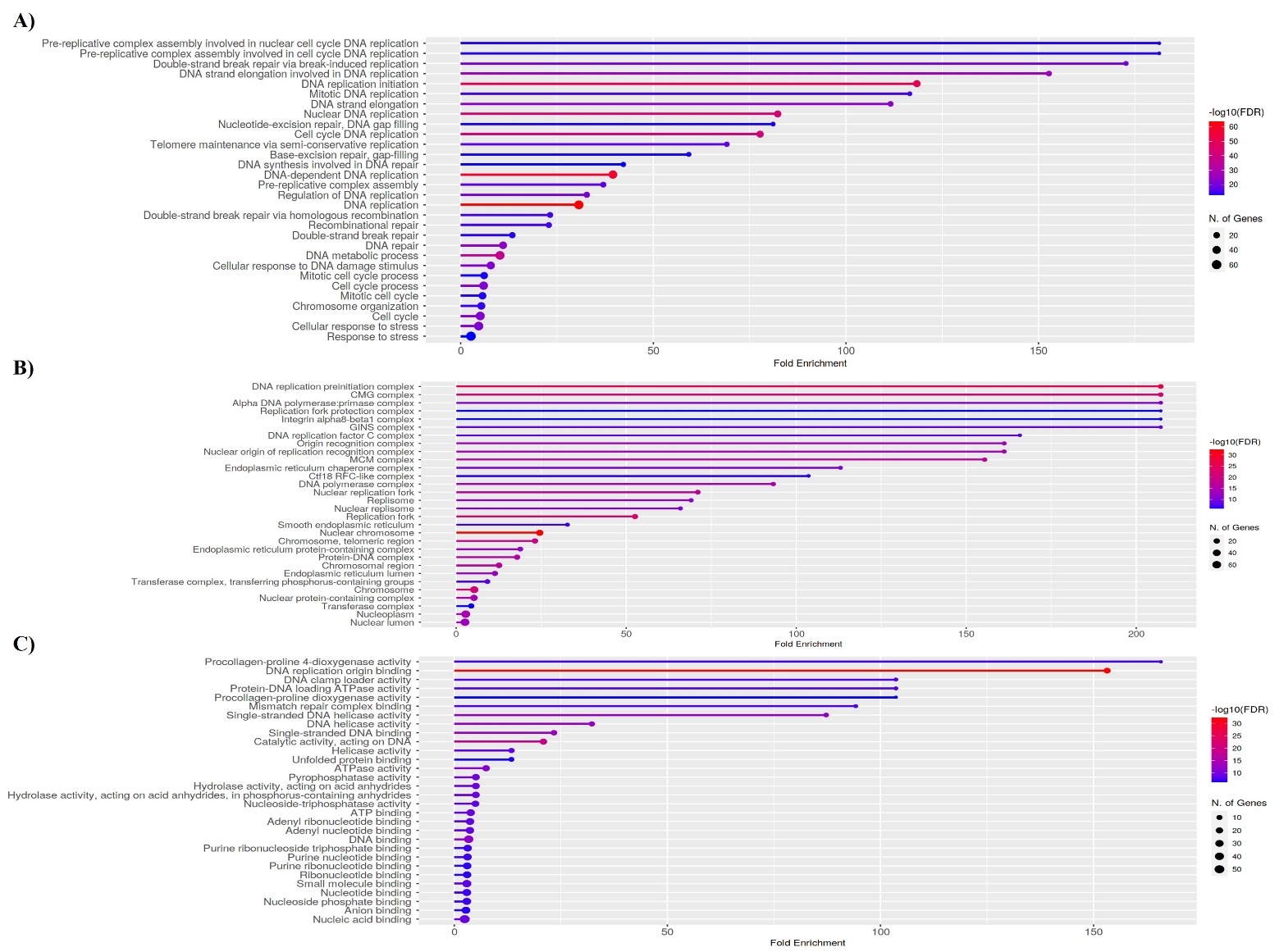


**Supplementary Figure S2.** Transcription factor enrichment analysis-top 20 transcription factors involved DEGs regulation


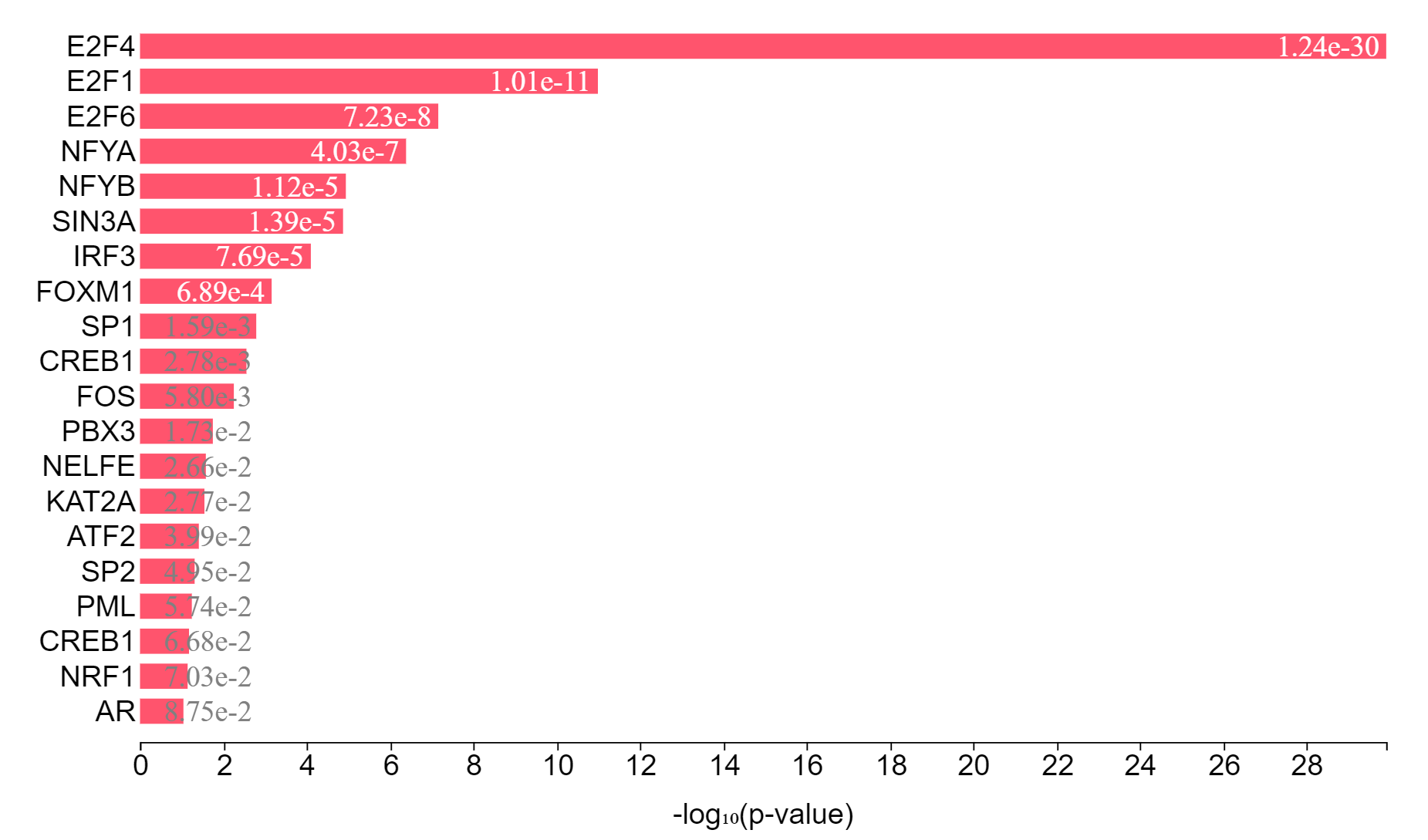


**Supplementary Figure S3.** The enrichment analysis of kinase with transcription factors and PPI network. A) Top 20 kinase is displayed in a bar graph from kinase-substrate interaction databases


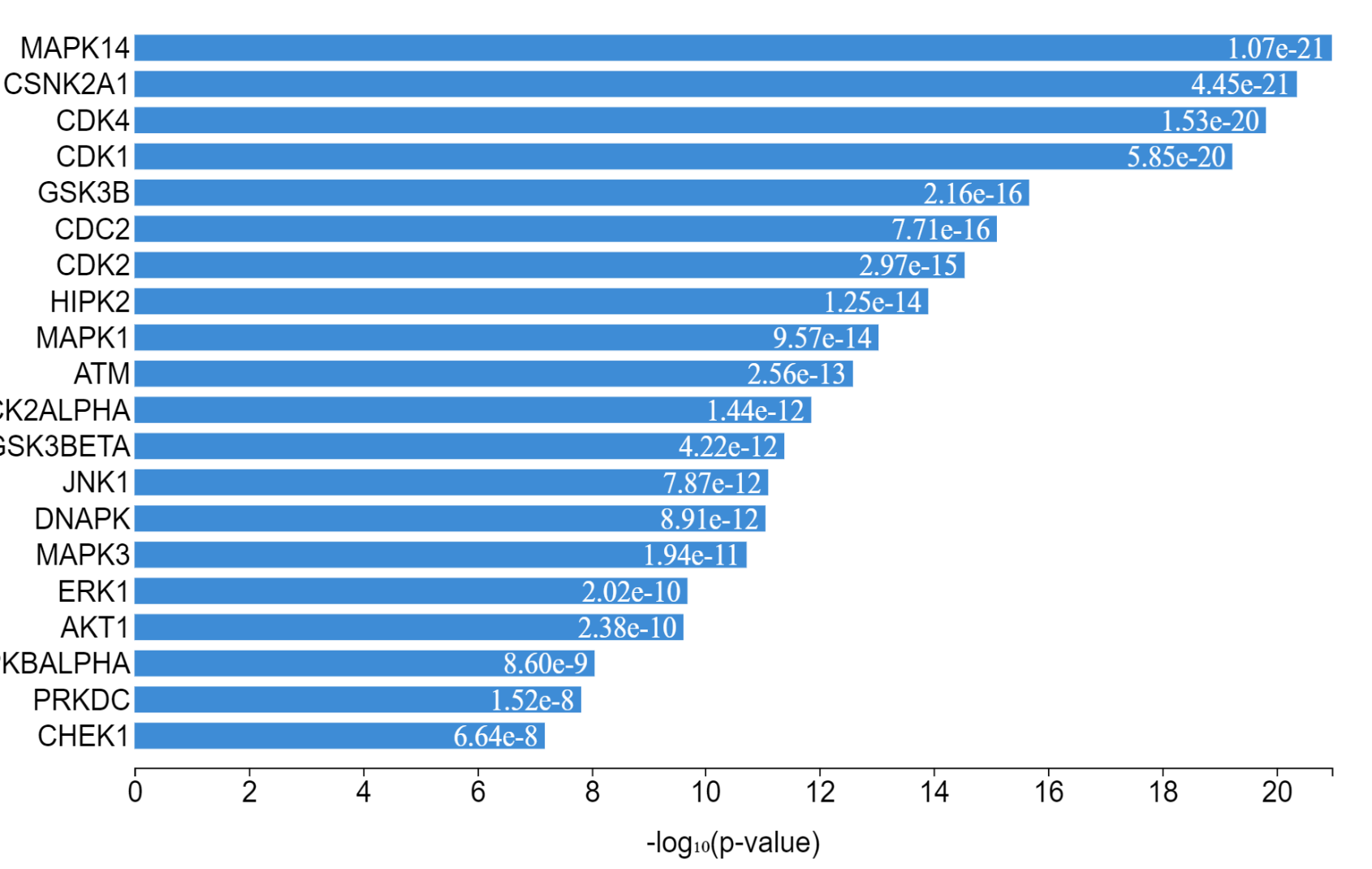


**Supplementary Figure S4.** The potential biochemical pathways associated with FG, CAG and GC were examined using FunRich software.


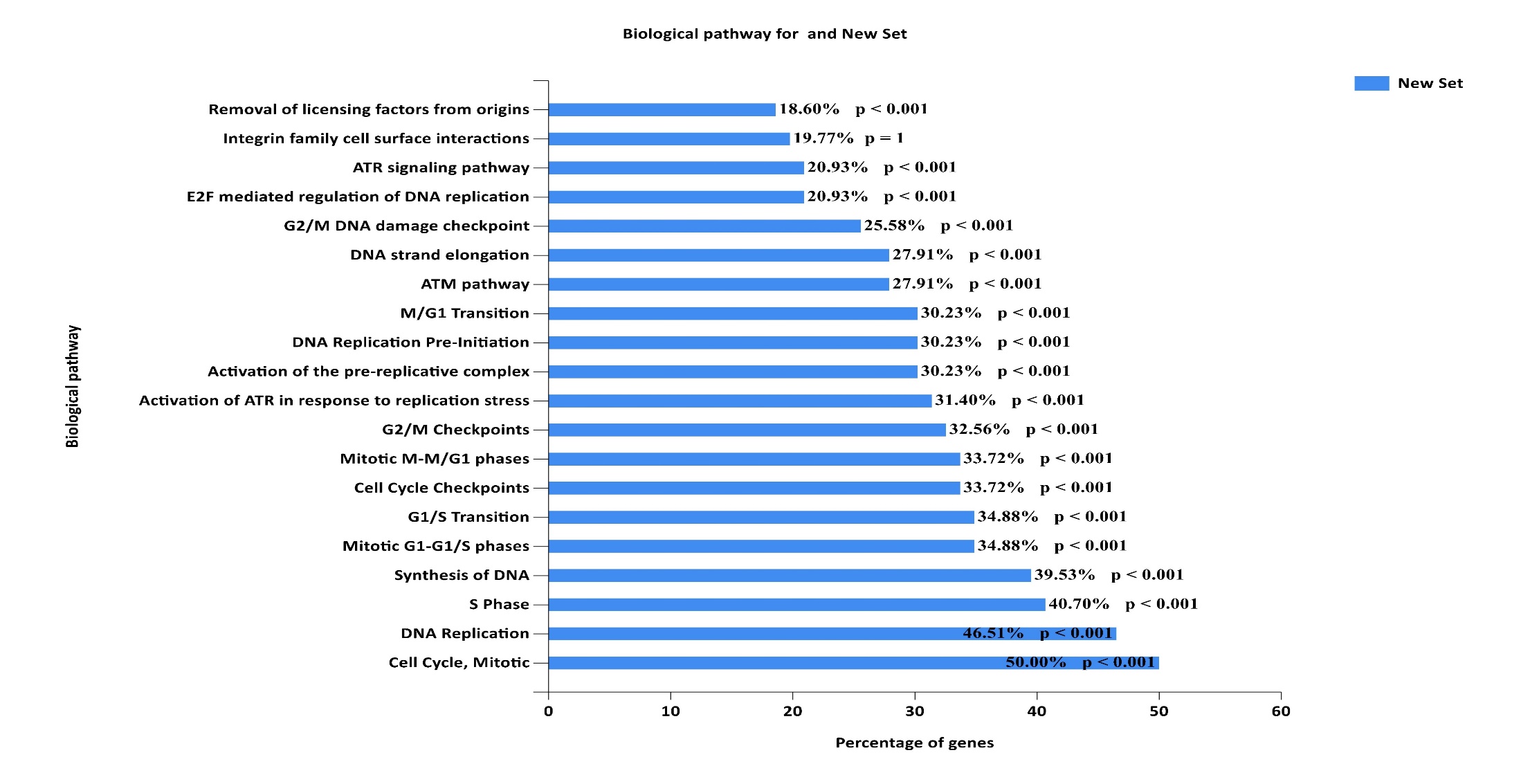


**Supplementary Figure S5.** Interactive survival scatter plot and survival analysis of the proteins (A) MCM7 and (B) CDC6


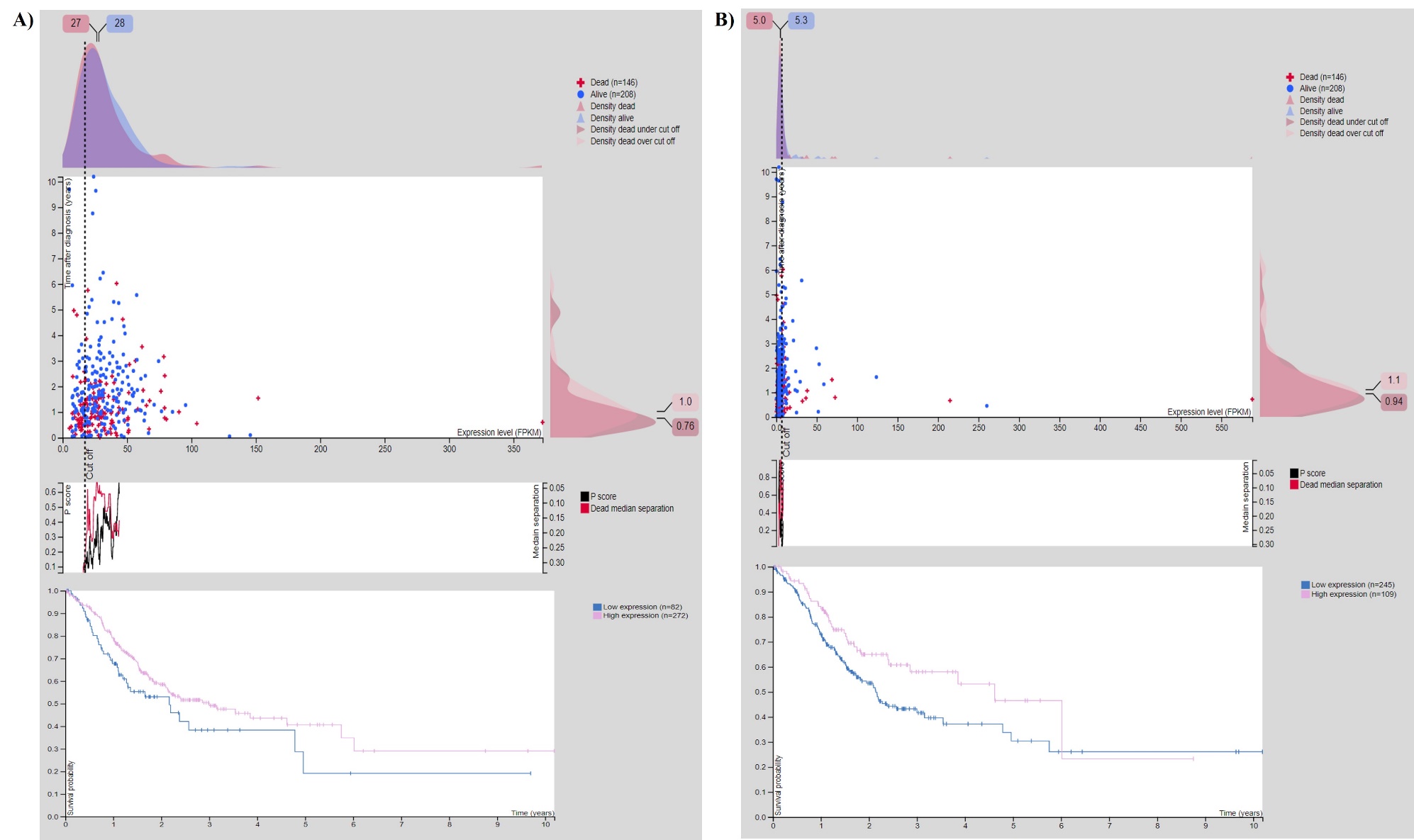

Supplement: Supplementary file 1 — Additional file 1. Supplementary materials. [file 43141_2023_539_MOESM1_ESM.docx]
